# HuR Plays a Role in Double-Strand Break Repair in Pancreatic Cancer Cells and Regulates Functional BRCA1-Associated-Ring-Domain-1(BARD1) Isoforms

**DOI:** 10.3390/cancers14071848

**Published:** 2022-04-06

**Authors:** Aditi Jain, Matthew McCoy, Carolyn Coats, Samantha Z. Brown, Sankar Addya, Carl Pelz, Rosalie C. Sears, Charles J. Yeo, Jonathan R. Brody

**Affiliations:** 1 The Jefferson Pancreas, Biliary and Related Cancer Center, Department of Surgery, Thomas Jefferson University, Philadelphia, PA 19107, USA; carolyn.coats@jefferson.edu (C.C.); samantha.brown@jefferson.edu (S.Z.B.); charles.yeo@jefferson.edu (C.J.Y.); 2 Sidney Kimmel Cancer Center, Thomas Jefferson University, Philadelphia, PA 19107, USA; sankar.addya@jefferson.edu; 3 Department of Oncology, Innovation Center for Biomedical Informatics, Georgetown University Medical Center, Washington, DC 20007, USA; matthew.mccoy@georgetown.edu; 4 The Department of Surgery, Brenden-Colson Center for Pancreatic Care, The Knight Cancer Institute, Oregon Health & Science University, Portland, OR 97201, USA; 5 The Department of Molecular and Medical Genetics, Brenden-Colson Center for Pancreatic Care, The Knight Cancer Institute, Oregon Health & Science University, Portland, OR 97201, USA; pelzc@ohsu.edu (C.P.); searsr@ohsu.edu (R.C.S.)

**Keywords:** pancreatic ductal adenocarcinoma, BARD1, HuR, RNA binding protein, DNA repair

## Abstract

**Simple Summary:**

RNA binding proteins (RBPs) post-transcriptionally regulate and stabilize a variety of target mRNA transcripts. Human Antigen R (HuR) binds to several pro-survival mRNA transcripts and promotes tumor cell survival and chemotherapeutic resistance. Some of these targets are within the DNA damage repair pathway that help cells repair damaged DNA and allow them to proliferate. Homologous recombination repair is the preferred method of DNA repair in the cells. Here, we aim to understand if HuR regulates homologous recombination repair in pancreatic ductal adenocarcinoma (PDAC). Our analysis, using Ribonucleo-protein Immunoprecipitation (RNP-IP) coupled with microarray and RNA-sequencing studies, revealed that HuR binds BARD1 (BRCA-1 Associated Ring Domain 1) and regulates expression of BARD1 mRNA full length transcripts and alternative isoforms. Silencing BARD1 mRNA potentiated the DNA damage effects of clinically relevant drugs, olaparib and oxaliplatin, in PDAC cells. Together, this work underscores a transient regulatory DNA repair pathway (i.e., HuR—BARD1 mRNA), which is likely a PDAC therapeutic resistance mechanism.

**Abstract:**

Human Antigen R (HuR/ELAVL1) is known to regulate stability of mRNAs involved in pancreatic ductal adenocarcinoma (PDAC) cell survival. Although several HuR targets are established, it is likely that many remain currently unknown. Here, we identified BARD1 mRNA as a novel target of HuR. Silencing HuR caused a >70% decrease in homologous recombination repair (HRR) efficiency as measured by the double-strand break repair (pDR-GFP reporter) assay. HuR-bound mRNAs extracted from RNP-immunoprecipitation and probed on a microarray, revealed a subset of HRR genes as putative HuR targets, including the BRCA1-Associated-Ring-Domain-1 (BARD1) (*p* < 0.005). BARD1 genetic alterations are infrequent in PDAC, and its context-dependent upregulation is poorly understood. Genetic silencing (siRNA and CRISPR knock-out) and pharmacological targeting of HuR inhibited both full length (FL) BARD1 and its functional isoforms (α, δ, Φ). Silencing BARD1 sensitized cells to olaparib and oxaliplatin; caused G2-M cell cycle arrest; and increased DNA-damage while decreasing HRR efficiency in cells. Exogenous overexpression of BARD1 in HuR-deficient cells partially rescued the HRR dysfunction, independent of an HuR pro-oncogenic function. Collectively, our findings demonstrate for the first time that BARD1 is a bona fide HuR target, which serves as an important regulatory point of the transient DNA-repair response in PDAC cells.

## 1. Introduction

Pancreatic ductal adenocarcinoma (PDAC) is the third leading cause of cancer-related deaths in the United States [1]. Although current therapies like FOLFIRINOX or gemcitabine-nab-paclitaxel continue to show modest success, prognosis for PDAC is still poor, with a five-year overall survival rate of only 11% [1,2,3]. Genome-wide sequencing analyses of PDAC tumors have shed light on the four high-frequency mutations (*KRAS*/*SMAD4*/*TP53*/*CDKN2A*) as well as the homologous recombination repair (HRR) pathway (e.g., *BRCA1*/*BRCA2* mutations and others) which all may set the stage for DNA repair defects, and thus, promote PDAC progression [4,5,6].

Roughly, 3–10% of all PDAC patients and 10–20% of high-risk Ashkenazi Jewish patients harbor either germline or somatic mutations in the *BRCA1*/*BRCA2* DNA repair genes [6,7]. For this subset of patients, as well as those carrying defects in other DNA damage repair (DDR) genes (Fanconi Anemia genes, *PALB2*, *ATM*), poly (adenosine diphosphate–ribose) polymerase inhibitors (PARPi) appear to have enhanced efficacy [6,8]. For instance, following promising results from the Phase III POLO trial of the PARP inhibitor, olaparib, in 2019, the FDA approved this drug for maintenance treatment of PDAC patients (*BRCA1*/*2* mutant tumors) who have not progressed on first-line platinum therapy [7], even though later results did not find an advantage to overall survival in these patients. Positive results were also reported from a Phase I/II trial involving a combination of veliparib (PARPi) and FOLFOX in patients with metastatic PDAC [9]; however, given the relative rarity of *BRCA*-defects in PDAC and resistance mechanisms, the benefits of this therapeutic strategy is amenable to only a limited number of patients, and the majority of patients who respond will eventually succumb to this disease. Hence, understanding DDR targets beyond *BRCA1*/*2* mutations and their mechanism of regulation is necessary to identify novel therapeutic opportunities for patients with PDAC.

Previously, we have demonstrated that the RNA-binding protein, Human Antigen R (HuR/*ELAVL1*) is upregulated in PDAC cells compared to normal cells [10,11,12,13,14]. HuR post-transcriptionally stabilizes mRNA and promotes upregulation of genes important for cell survival and proliferation [10,11,15,16]. In response to DNA-damaging agents, HuR stabilizes the mitotic kinase inhibitor, WEE-1, to attenuate cell-cycle progression and facilitate damage-induced repair [16]. Moreover, silencing HuR increases drug-induced DNA damage, impairs cell-cycle progression, and increases efficacy of chemotherapeutic agents [10,16]. Recent studies have also shown the importance of the post-transcriptional regulation of DDR genes by HuR in other cancer types [17]. HuR also enhances the p53 translation and mRNA stability of p21 in the presence of UV light [18,19]. Collectively, these studies point to an important regulatory role that HuR plays in the DDR response. Using an established double-strand-break repair assay (pDR-GFP), we demonstrate for the first time that HuR directly affects HRR activity in PDAC cells [20,21,22]. At the protein level, HuR carries three RNA-recognition motifs (RRM) with a high affinity for U- and AU-Rich Elements (AREs), which are most prevalently embedded in the 3′-untranslated region (3′UTR) of target mRNAs. HuR binds to these unique sequences, and as a result, target mRNAs are either stabilized, or prevented from degradation by microRNAs (miRNAs) that bind to similar ARE sequences. These changes increase the stabilization of mRNA, which increases protein levels [12,23,24,25]. In some instances, AREs have been reported in intronic regions, where HuR could participate in mRNA processing and/or splicing [13,26,27]. Through a whole-transcriptome ribonucleoprotein immunoprecipitation (RNP-IP) microarray, we identified that HuR uniquely regulates BRCA1-Associated-Ring-Domain-1 (BARD1), mRNA abundance in PDAC cells, and its pre-mRNA levels, as well as different functional isoforms of BARD1 (α, δ, Φ) mRNA. 

BARD1 is an obligatory partner of BRCA1 and participates in the homologous recombination repair of DNA double-strand breaks by heterodimerizing with BRCA1 via its RING domain [28,29,30]. Independent of BRCA1 status, BARD1-null mice show early embryonic lethality and die between embryonic day 7.5 and 8.5, which suggests that BARD1 is an independent promoter of genomic stability, essential for cell survival and proliferation of cells through its DNA repair activity [31]. Multiple splice variants or functional isoforms of BARD1 comprising of variable exon composition are expressed in human and murine cancers. Many of these BARD1 isoforms lack the RING finger, which is required for BRCA1 interaction [32,33,34]. At least 11 isoforms are protein coding and have been described in the literature. As early as 2004, the role of BARD1 isoforms was described in the process of spermatogenesis [35]. Later, many functional isoforms have shown tumorigenic potential in different cancer types, as well as in their ability to enhance proliferation and invasion of human cytotrophoblasts [32,33,36,37,38]. However, exact cellular functions of these isoforms remain unclear. There is some evidence, for example, that the elevated expression of the BARD1 β isoform has shown to be an oncogenic driver for high-risk neuroblastoma [39]. Similarly, BARD1 δ is expressed in many cancers and its expression correlates with highly aggressive clear cell ovarian cancer and other gynecological cancers [32,33,39,40]. Isoform Φ was also found in tumors with poor outcomes in ovarian and breast cancer patients [33]. Although expressed, isoforms δ and Φ were found to have an inhibitory effect on the tumorigenesis of colon cancer in one study [40]. Thus, the role of these isoforms remains unclear, and their functions are most likely dependent on the cell type and setting. 

We suggest that PDAC tumor cells may utilize the HuR-BARD1 axis to maintain the integrity of DNA repair and cellular proliferation under conditions of DNA damage exposure, such as chemotherapy.

## 2. Materials and Methods

### 2.1. Cell Culture

Pancreatic adenocarcinoma cells (PDAC) MiaPaCa-2, Panc-1, and AsPC-1 were obtained from the American Type Culture Collection (Manassas, VA, USA) and maintained in a culture according to the manufacturer’s instructions. All cell lines were cultured in DMEM with a 10% FBS at 5% CO_2_ and at 37 °C. All cell lines were STR authenticated and were tested for mycoplasma monthly, using a PCR-based mycoplasma detection kit (#MP0035 Sigma Aldrich, St. Louis, MO, USA). Cells were passaged at least twice after thawing before experimental use. Stable MiaPaCa-2-DRGFP cells were generated after the transfection of cells with pDR-GFP (#26475 Addgene, Watertown, MA, USA), selecting cells with puromycin (10 µg/mL), and single sorting using BD FACS Aria sorter.

### 2.2. Drugs

Olaparib (PARP inhibitor) and oxaliplatin were obtained from MedChem Express (Monmouth Junction, NJ, USA). Gemcitabine and actinomycin D were obtained from Sigma Aldrich. Stock solutions were diluted prior to use at indicated concentrations.

### 2.3. Plasmid and siRNA Transfections

siRNA transfections were carried out using RNAiMAX (Thermo Fisher Scientific, Waltham, MA, USA) for 48 h according to the manufacturer’s protocol. The following siRNAs were purchased from Thermo Fisher Scientific: siBARD1#1:s1885 (5′-GCCUGUCGAUUAUACAGAUTT-3′), siBARD1#2:s1887 (5′-CGCUAUUGCUGCUACCAGATT-3′), and siIDH-1: 107867. For HuR silencing, customized siRNA oligonucleotide (5′-AAACCAUUAAGGUGUCGUAUGUU-3′, siHuR#1) and SMARTpool siRNA (siHuR#2: L-003773-00-0005) were purchased from Horizon Discovery Ltd. (Cambridge, UK) and used as previously described [11]. Non-targeting siRNA (siSCR) (D-001810-01-20), siBRCA2 (L-003462-00-0005) were from Horizon Discovery Ltd. Full length (FL) BARD1 was purchased from Genscript (Piscataway, NJ, USA). All plasmid transfections were done using a Lipofectamine 2000 reagent at 1:3 (DNA: Lipofectamine) for 48 h and was used accordingly. 

### 2.4. RT-qPCR and mRNA Expression Analysis 

Total RNA was extracted using the RNeasy mini kit (Qiagen Inc., Germantown, MD, USA). cDNA was made using 2 μg total RNA using Applied Biosystems High Capacity cDNA Reverse Transcriptase kit (Life Technologies Corp, Carlsbad, CA, USA) and quantitative PCR (RT-qPCR) was performed as previously described [41,42]. Relative quantification was performed using the 2-ΔΔCt method. Specific forward and reverse primers for RT-qPCR analysis were: 5′-GCTTAATTTGACTCAACACGGGA-3′ and 5′-AGCTATCAATCTCTCAAGCCTGTC-3′ for 18S; 5′-CATGCGAGACCCGATTCTGA-3′ and 5′-TAAACCAGCTCGAAGGAGCC-3′ for BARD1; 5′-GCTCGGTCTACTCAGGCATC-3′ and 5′-CCAGTCCAGGAGCCTAATGA-3′ for HuR; intron–exon primers for BARD1 used were:- 5′-AGGGCGACATACCTTCTGTT-3′ and 5′-AAAGAGTATATGTGGCAGAGGATGA-3′ for int-ex-5; 5′-CCTCTGCTCCATTTATTTCTGTTCA-3′ and 5′-CAAGTCTTGTATCCAGGCCG-3′ for int-ex-3 (pair 1); 5′-ATTAAATTCTGCTGAATGGGTTGC-3′ and 5′-TATCCAGGCCGGGGTGTAAC-3′ for int-ex-3 (pair 2); intron–exon primers for GAPDH used were:—5′-GCTCCCTTGGGTATATGGTAAC-3′ and 5′-TTCTCCATGGTGGTGAAGAC-3′ for int-ex-5; 5′-GGAAATCCCATCACCATCTTCC-3′ and 5′-ATATACCCAAGGGAGCCACA-3′ for int-ex-4; isoform specific primers used were as described in [34]:—5′-TGCTCGCGTTGTAATTGTGT-3′ and 5′-TATCCAGGCCGGGGTGTAA-3′ for alpha, 5′-GCTGCTCGCGTTGATTTGA-3′ and 5′-CGAGGGCTAAACCACATTTTAATT-3′ for beta, 5′-TGAGCTGTCAGGGCGACATACC-3′ and 5′-TTCCACTACCTTCAGGTGCCCA-3′ for gamma, 5′-CGCTGCTCGCGTTGTAATATATTTGGT-3′ and 5′-CCCTACGCTGCCCAGTGTTCA-3′ for delta, 5′-GTGAGCACATCTTCTGTAGTAATATATTTGGT-3′ and 5′-CCCTACGCTGCCCAGTGTT-3′ for phi.

### 2.5. Analysis of BARD1 mRNA Stability in HuR-Silenced Cells by Actinomycin D 

MiaPaCa-2 cells were plated in 6-well plates and transfected with either siScramble (siSCR) or siHuR (siHuR#1). Twenty-four hours later, cells were treated with actinomycin D (5 µg/mL) in the cell culture medium and RNA was isolated at indicated time points (0 to 8 h). The relative amount of specific mRNA remaining in each sample was correlated with mRNA degradation. BARD1 mRNA half-lives were calculated from decay curves by linear regression between 0 h and 8 h [15,16,17]. Results are representative of three independent experiments.

### 2.6. Ribonucleo-Protein Immunoprecipitation (RNP-IP) Assay

MiaPaCa-2 cells at 70% confluence were treated either with olaparib (10 µM), oxaliplatin (5 µM), or gemcitabine (1 µM) for 16 h, washed with 1× phosphate buffered saline (PBS), harvested using cell scraper solution, and fractionated using a NEPER nucleo-cytoplasmic fractionation kit (Thermo Fisher Scientific). Cytoplasmic lysates were subjected to immunoprecipitation using either 30 μg of an anti-HuR antibody (the RNP-IP specific antibody was purchased from MBL International, Woburn, MA, USA) or immunoglobulin (IgG) control as previously reported [16,43]. RNA isolated from immunoprecipitation were subjected to RT-qPCR analysis or microarray using human Clariom D assay (Thermo Fisher Scientific). Validation of nuclear, cytoplasmic fractions and HuR-IP were done using western blot analysis.

### 2.7. Microarray and Bioinformatics Analysis (GEO Accession # GSE166951)

Fragmented biotin labeled cDNA (from 10 ng of RNA) was synthesized using an Affymetrix GeneChip WT Pico kit. RNA from HuR RNP-IP was used to generate cRNAs, and those cRNAs were then used to generate cDNAs. Affymetrix gene chips, Human Clariom D array, was hybridized with a fragmented and biotin-labeled target (5 µg) in 220 µL of a hybridization cocktail. Target denaturation was performed at 99 °C for 5 min and then 45 °C for 5 min, followed by hybridization for 16–18 h. Raw probe intensities from the Affymetrix CEL files were preprocessed and normalized into CHP formatted files using Expression Console Software 1.4 (Life Technologies Corp, Carlsbad, CA, USA) and extracted using the affxparser library in R (https://github.com/HenrikBengtsson/affxparser, accessed on 18 January 2018). Probe signals were further processed by determining the average HuR intensity relative to the IgG signal to identify transcripts that are enriched for HuR binding for all treatment conditions. Differential HuR interactions were determined by fitting a linear model to the normalized HuR intensities to identify differentially bound transcripts using Limma [44]. GEO Accession # GSE166951.

### 2.8. Gene Set Enrichment Analysis of siHuR vs. siSCR RNA-seq Database (GEO Accession # GSE167525)

Gene Set Enrichment analysis (GSEA, version 4.0, Broad Institute, Cambridge, MA, USA) was used to analyze DNA repair gene sets (Reactome group from the Molecular Signatures Database, MSigDB, ver. 6.0, accessed on 4 June 2021) from the RNA-Seq database of siHuR (HuR_NT) versus siScramble (CTRL_NT) (GEO Accession # GSE167525). We selected 1000 permutations and a false discovery rate (FDR ≤ 0.25); all other features were set to default settings.

### 2.9. Comet Assay

Alkaline comet assay was performed using the Comet Assay Kit (Trevigen, Gaithersburg, MD, USA) according to the manufacturer. MiaPaCa-2 cells were seeded in 6 well plates. Twenty-four hours later, cells were exposed to the appropriate treatment groups. After treatment, cells were harvested, washed with ice-cold 1× PBS, and re-suspended in a 1% low-melting agarose that was supplied by the manufacturer. The agarose/cell mixture was spread onto glass comet slides and allowed to solidify. The slides were then immersed in an ice-cold lysis buffer at 4 °C overnight. After lysis, the slides were electrophoresed in a cold 1× TBE buffer for 30 min using a electrophoresis system used for DNA gels, fixed with 70% ethanol, and stained with SYBR gold for 30 min in the dark. Comet images were captured using a Leica microscope and tail DNA percentage was measured with a minimum of 30–50 comets per sample using the Open comet software through ImageJ in each experiment as described by Gyori et al. [45] H_2_O_2_ treated cells were used as positive controls. This assay was repeated three times and an average percentage of Tail DNA was plotted.

### 2.10. pDR-GFP Assay to Assess HRR

The GFP-based double strand break repair assays were performed as described previously [21,22] with modifications. MiaPaCa-2 cells were transfected with the pDR-GFP plasmid (Addgene plasmid #26475) and selected with puromycin. MiaPaCa-2-DRGFP cell lines were plated in 10 cm dishes and first transfected with siBARD1 (siBARD1#1), siHuR (siHuR#1), or siScramble (siSCR) siRNAs using a Lipofectamine RNAiMAX reagent. Twenty-four hours later, cells were again transfected with 10 µg pCBASceI (Addgene plasmid# 26477) or with control plasmids using a Lipofectamine 2000 reagent. Ninety-six hours after transfection, an equal number of cells were collected, and the percentages of GFP-positive cells were quantitated (as a readout of HRR efficiency) by flow-cytometry after staining cells with propidium iodide dye to exclude dead cells.

### 2.11. Cell Survival and Colony Formation

Cell survival was analyzed using a Pico Green assay as previously described [42]. The percentage of relative cell survival was calculated and plotted using GraphPad Prism 8.4.3 software (GraphPad, San Diego, CA, USA). Colony formation assays were conducted in 6-well plates. Five hundred to one thousand cells were plated in each well and were allowed to adhere for 24 h before drug treatments. Cells were treated every 72 h and colonies were allowed to form for 14 days [42,45]. Colonies were fixed in methanol and stained with freshly prepared Coomassie blue solution (0.05% Coomassie blue in 80% methanol and 20% water).

### 2.12. Immunoblot Analysis 

Cells were lysed in an ice-cold RIPA buffer (Santa Cruz Biotechnology Inc., Dallas, TX, USA) supplemented with a fresh protease inhibitor cocktail, PMSF, and phosphatase inhibitor sodium orthovanadate. Protein samples were made in 5× Laemlli buffer and fractionated on SDS-PAGE gels (Bio-Rad, Hercules, CA, USA). Samples were transferred on PVDF membranes and probed with primary and secondary antibodies, before scanning and quantitation using the Odyssey Infrared Imaging System (LI-COR Biosciences, Lincoln, NE, USA) as previously described [41,42] (the original blots see Figure S7). Primary antibodies to HuR and α-Tubulin were purchased from Thermo Fisher Scientific; Lamin A/C, Cyclin-B1, Cdk-1 and p-Cdk-1 (tyr15) were purchased from Cell Signaling Technology (Danvers, MA, USA); the BARD1 antibody was purchased from Santa Cruz Biotechnology Inc.

### 2.13. Immunofluorescence

BARD1 silenced or scramble control cells were plated onto coverslips in 24-well tissue culture plates and allowed to adhere for 24 h. Cells were treated for 24 h, coverslips were washed with 1× PBS, fixed (4% paraformaldehyde for 10 min RT), permeabilized (0.1% Triton-X), blocked, and immunostained with a γ-H2AX 1:500 (Millipore Sigma, Burlington, MA, USA) antibody overnight, at 4 °C, followed by a secondary antibody (Alexa Fluor 488 anti-mouse, Thermo Fisher Scientific) for 1 h after washing. After the final wash step, coverslips were mounted onto slides using a DAPI Pro-Long Gold anti-fade mounting medium and they were imaged with a Leica DM4B fluorescence microscope. γ-H2AX foci were counted using Image J software (NIH, Bethesda, MA, USA) [10,15]. The percentage of cells expressing > 10 γ-H2AX foci per treatment condition ± SEM was calculated and plotted [42].

### 2.14. Cell Cycle Analysis

Cells were harvested, counted using a hemocytometer, and re-suspended in 1× PBS. Cells in PBS were fixed in ice-cold 70% ethanol at −20 °C for at least 24 h. Cells were then washed twice with 1× PBS, treated with RNAse A (10 µg/mL) for 20 min at room temperature, washed with PBS, and treated with propidium iodide (2 µg/mL) for at least 30 min at room temperature. Samples were analyzed using the Millipore Guava Easycyte and cell cycle analysis was performed via GuavaSoft 3.1.1 software (Millipore Sigma, Burlington, MA, USA) [41].

### 2.15. BARD1 mRNA Expression and Survival Analysis 

Oncomine^TM^ datasets (www.oncomine.org, accessed on 5 January 2020) were used to analyze BARD1 expression in PDAC versus normal pancreas. From the Logsdon Pancreas dataset, we compared primary tumor samples (*n* = 10) and PDAC cell lines (*n* = 7) against normal pancreas samples (*n* = 5), which we analyzed using cDNA microarrays [46]. The Badea Pancreas dataset compared larger cohorts with PDAC (*n* = 39) and adjacent normal pancreas specimens (*n* = 39) [47]. mRNA levels of BARD1 in tumor tissue were compared to the normal group using Student’s *t*-test to generate *p*-value (following default settings of Oncomine). 

Kaplan–Meier survival plots for BARD1 in PDAC were obtained from the online database, KM plotter [48]. Hazard ratio (HR) and 95% confidence intervals (CI), as well as log rank P, are indicated on the plot. A *p* value of <0.05 was considered to be statistically significant. HR is the ratio of the hazard rates that correspond to the conditions described by two levels of an explanatory variable in survival analysis.

### 2.16. Statistical Analysis

Statistical analyses were performed using the Student’s *t*-test or one-way ANOVA. GraphPad Prism v.8.4.3 software (GraphPad, San Diego, CA, USA) was used for analysis. Results are expressed as mean ± SEM, if not otherwise indicated.

## 3. Results

### 3.1. HuR Regulates Homologous Recombination Repair in PDAC Cells

**Figure 1:**
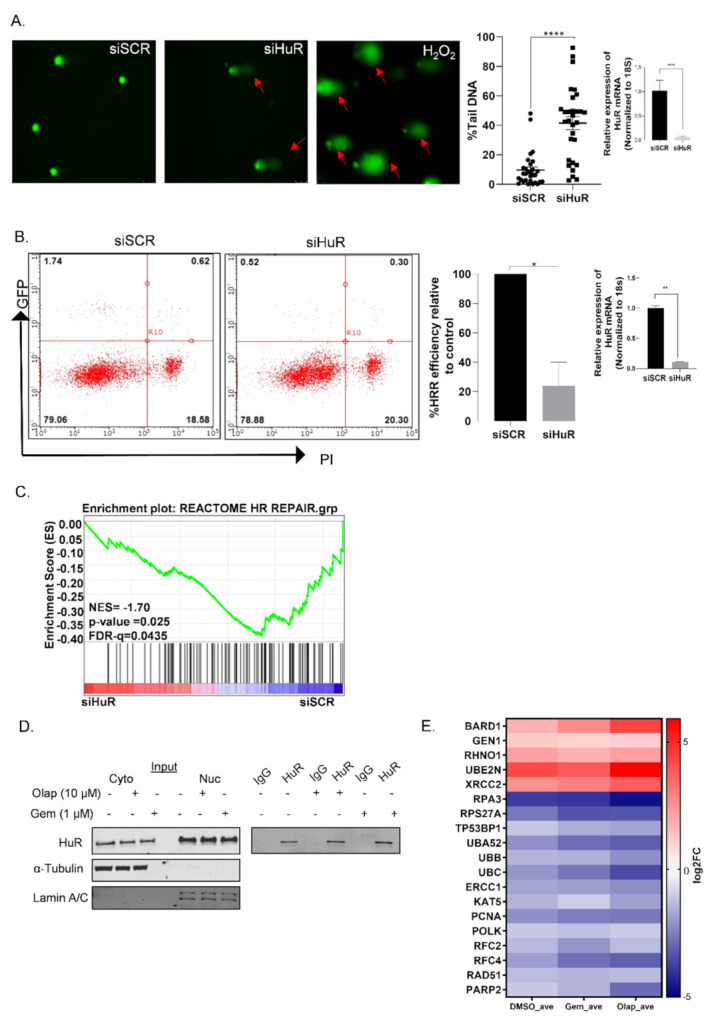
HuR regulates homologous recombination repair in PDAC cells. (**A**) Comet assay showing an increase in DNA damage when MiaPaCa-2 cells were transfected with siHuR (siHuR#1) as compared with the siSCR control. H_2_O_2_ was used as a positive control (the scale bar: 10–50 μM). Mean ± SEM, *n* = 3. **** *p* < 0.0001 (Student’s *t*-test). Validation of HuR knockdown is shown on the right. (**B**) pDR-GFP assay: MiaPaCa-2-DRGFP cells were transfected with siHuR (siHuR#1) and 24 h later they were re-transfected with pCBASceI (10 µg). Graph shows the percentage of HRR efficiency by comparing siHuR and siSCR, as calculated by quantitating GFP-positive cells using flow cytometry. Mean ± SEM, *n* = 3. * *p* < 0.05, ** *p* < 0.01 (Student’s *t*-test). Validation of HuR knockdown is shown on the right. (**C**) Gene set enrichment analysis of siHuR (HuR_NT) vs. siSCR (CTRL_NT) (*n* = 4) samples in MiaPaCa-2 cells, showing negative enrichment of the Reactome homologous recombination repair pathway. (**D**) Western blot showing validation of HuR fractions in cytoplasmic and nuclear compartments; and HuR-IP in MiaPaCa-2 cells. α-Tubulin was used as a control for cytoplasmic extracts and Lamin A/C was used as a control for nuclear extracts. (**E**) The Human Clariom D microarray was performed from HuR-RNP-IP MiaPaCa-2 samples and was treated with either olaparib (10 µM) or gemcitabine (1 µM) for 16 h. A heatmap of log2FC is shown of the genes from the Reactome homologous recombination repair pathway from two sets for each treatment condition (HuR vs. IgG).

We have previously shown that an inhibition of HuR in stress conditions is associated with DNA damage [10,16]. We validated the direct effect of HuR silencing (using two different siRNAs, siHuR#1 and siHuR#2) on DNA damage by performing comet assays in MiaPaCa-2 cells; H_2_O_2_ was used as a positive control for inducing DNA damage [49]. We found that HuR loss increases the accumulation of damaged DNA in cells compared with siSCR control cells, as assessed by percentage tail DNA (Figure 1A and Figure S1a). Next, we used a well-established HRR efficiency assay, originally developed by the Jasin lab. (MSKCC, New York, NY, USA) [21,22]. This direct-repeat GFP (pDR-GFP) reporter construct encodes two incomplete halves of a GFP cassette with a premature stop codon in-between. Upon transfection with I-SceI endonuclease, this early stop site is removed, and HRR efficiency can be evaluated as percent of GFP positivity. With this assay, we showed that the loss of HuR decreased GFP+ expressing cells by >70% (*p* value = 0.0416) compared with the control cells (Figure 1B). Positive (siBRCA2) or negative (siIDH1) controls for the pDR-GFP assay are presented in Figure S1b. These data suggest that HuR participates, either directly or indirectly via its targets, in the homologous recombination repair mechanism of cells. 

### 3.2. A Subset of DDR Genes Are Targets of HuR: BARD1 Is a Novel HuR Target

To follow up on these results, we aimed to determine whether HuR directly affects genes from the DNA repair and/or HRR pathway. We examined gene sets from the Molecular Signatures Database (MSigDB, Broad Institute) and performed gene set enrichment analysis (GSEA) on RNA-seq samples transfected with siHuR (HuR_NT), as compared with siSCR (CTRL_NT) (Geo Accession # GSE167525). The data demonstrated a strong de-enrichment of the “Reactome of HR Repair” within siHuR samples (FDR ≤ 0.25, Figure 1C and Figure S1c,d). The details of GSEA are provided in Supplementary Table S1. Since the loss of HuR may be contributing to both direct and indirect effects on this pathway, we investigated what members of the pathway are direct HuR targets, by using RNP-IP assays [10,16,43]. Although HuR is shown to be a nuclear protein in normal cellular conditions, its functional activity is dependent on the shuttling event from nucleus to cytoplasm, particularly in conditions of stress (chemotherapy, low glucose, low nutrient etc.) and this cytoplasmic HuR is known to stabilize its mRNA targets (in the cytoplasm) and increase translation of target mRNAs [12,13,23,24,25]. Our lab has previously shown that cytoplasmic HuR correlates with higher tumor staging in PDAC [14]; therefore, we routinely collect cytoplasmic fractions where HuR translocates to, and interacts with, transcribed mRNA targets. PDAC cells were treated with an acute dose of previously identified stressors, olaparib or gemcitabine [10,15,16]. HuR was then immunoprecipitated from cytoplasmic fractions (Figure 1D), and HuR-bound RNA was run on a whole transcriptome Human Clariom D microarray (Geo Accession # GSE166951). Bioinformatics analyses indicated a differential expression of genes between HuR vs. IgG pulldown (*p* value < 0.05, all transcripts mapped in HuR vs. IgG are presented in Supplementary Table S2). We compared significant HuR-bound targets to those encompassing the ‘Reactome HR repair’ gene set from MSigDB and found that HuR binds a subset of genes that participate in the process of HRR (heat-map in Figure 1E). Publicly available databases from HuR RIP-chip or PAR-CLIP studies have cited putative HuR binding sites in some of these genes in other cancer types [26,50], thus supporting our findings in PDAC. BARD1 was significantly enriched (*p* value < 0.05) in HuR IP vs. IgG IP in all treatment conditions and had the highest log2FC differences between control (DMSO, 1.76 log2FC) and treated (olaparib, 4.32 log2FC; gemcitabine, 2.67 log2FC) conditions when compared with log2FC in *POLK*, *RHNO1*, *RAD51* or *PARP2*. All four of these genes were more significantly repressed in the RNA-Seq of siHuR (Supplementary Tables S2 and S3). However, *POLK*, *RAD51*, *PARP2* had no binding affinity to HuR in the RNP-IP assay and *RHNO1* had weak or no significant log2FC across treatment conditions, suggesting that their downregulation upon HuR knockdown is an indirect consequence of loss of HuR (Figure S1c,d). 

BARD1 is an obligatory partner of BRCA1. In a complex formation with BRCA1, BARD1 participates in the homologous repair of DNA double-strand breaks [28,29,30]. Independent of BRCA1, BARD1 is important in promoting genomic stability and is essential for cell survival and proliferation of cells [30,31]. Recent genomic screenings have found pathogenic and germline BARD1 mutations in PDAC patients which contributes to HRR deficiency; however, these incidences are very low (0.1–0.8%) [51,52,53], suggesting genetic alterations in BARD1 cannot account for its mRNA abundance in PDAC cells. In fact, in two independent datasets from the cancer microarray database, ONCOMINE (www.oncomine.org, accessed on 5 January 2020), we compared BARD1 expression levels in the primary tumor versus in normal pancreas samples [32,33]. Both datasets, Badea (*p* < 0.0001) and Logsdon (*p* = 0.0086), demonstrated a significant overexpression of BARD1 mRNA in PDAC tumors as compared with controls (i.e., normal tissue) (Figure S1e). Moreover, Kaplan–Meier survival curves in patients with PDAC, who were stratified based upon BARD1 expression, demonstrate that high BARD1 expression is an unfavorable prognostic factor for overall survival (HR = 1.59, *p* = 0.028) (Figure S1f). Hence, these results strengthened our initial findings and prompted us to focus on elucidating BARD1 as an important HRR protein and a target of HuR in PDAC. 

### 3.3. HuR Regulates mRNA Expression of BARD1

**Figure 2:**
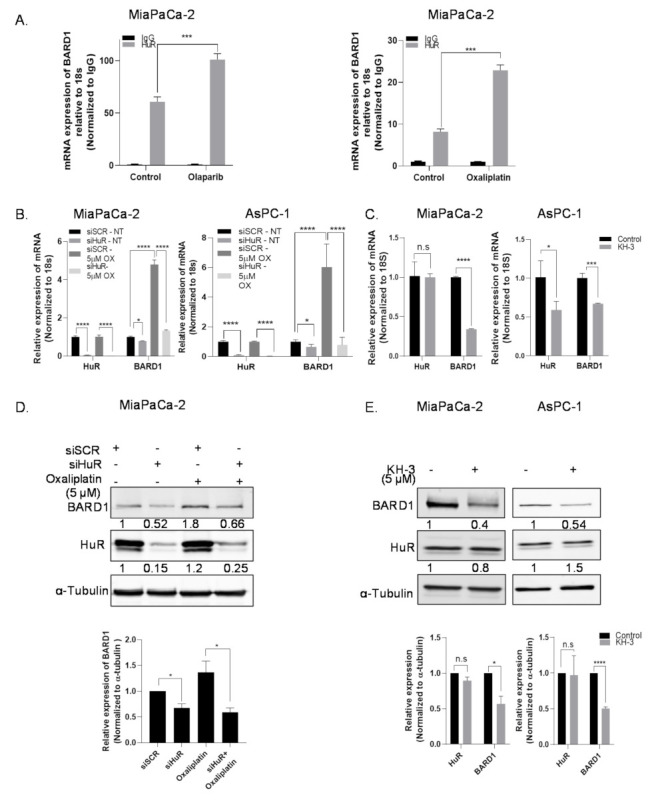
HuR regulates mRNA and protein expression of BARD1. (**A**) Independent HuR-RNP-IP was performed after treating cells with either olaparib (10 µM) or oxaliplatin (5 µM) and BARD1 mRNA expression was calculated relative to 18 s by RT-qPCR. Mean ± SEM, *n* = 3. *** *p* < 0.001 (Student’s *t*-test). (**B**) Relative mRNA expression of BARD1 in MiaPaCa-2 and AsPC-1 PDAC cells after treatment of cells with oxaliplatin and siHuR (siHuR#1) transfection. * *p* < 0.05, **** *p* < 0.0001. (**C**) Relative mRNA expression of BARD1 in MiaPaCa-2 and AsPC-1 cells treated with KH-3 (5 µM) for 48 h. Mean ± SEM, *n* = 3. *** *p* < 0.001, * *p* < 0.05, n.s, non-significant. (Student’s *t*-test). (**D**,**E**) Western blot analysis of the protein expression of BARD1, HuR, and α-tubulin in MiaPaCa-2 cells treated with oxaliplatin (5 µM) and transfected with siHuR (siHuR#1) for 48 h or treated with KH-3 for 72 h. Representative blots and graphs showing relative band intensities is shown. Mean ± SEM from *n* = 3, * *p* < 0.05, **** *p* < 0.0001, n.s, non-significant. (Student’s *t*-test).

To validate our RNP-IP-microarray findings, we performed independent HuR-RNP-IP in PDAC cell lines treated with or without chemotherapeutic stress [15,16]. Again, BARD1 was greatly enriched in HuR-IP over IgG control, which could be enhanced with olaparib or oxaliplatin treatments (Figure 2A). Validation of IP is shown in Supplementary Figure S2a. These data corroborate our microarray findings and confirm the direct interaction of HuR protein to BARD1 mRNA.

To determine the impact of this direct binding, we silenced HuR in PDAC cells and evaluated changes in total BARD1 mRNA expression. HuR silencing decreased mRNA expression of BARD1 in both MiaPaCa-2 and AsPC-1 cell lines; treatment of cells with a DNA-damage agent (e.g., oxaliplatin, 5 µM) induced a significant increase in the mRNA expression of BARD1, which could be significantly ablated when HuR was silenced (Figure 2B). BARD1 mRNA expression was also significantly downregulated in a knockout (KO) clone for HuR in MiaPaCa-2 cells, Mia (−/−), which was previously generated by CRISPR-mediated homozygous deletion [41] (Figure S2b). Furthermore, pharmacological inhibition of HuR using the specific small-molecule inhibitor KH-3 (a generous gift from Dr. Liang Xu’s lab., University of Kansas, Lawrence, KS, USA) [54], which disrupts HuR-mRNA interaction, also reduced BARD1 mRNA expression in both MiaPaCa-2 and AsPC-1 cell lines (Figure 2C). Downregulation of HuR mRNA levels, as seen in the AsPC-1 cell line upon treatment with KH-3, could be an off-target or cell line dependent effect.

### 3.4. HuR Impacts Total Protein Expression of BARD1

Post-transcriptional regulation by HuR often impacts translation of target mRNAs, resulting in abundance of protein expression [10,11,13,16,19,50]; therefore, we evaluated if HuR regulates BARD1 protein expression in PDAC cells. Western blot analysis of MiaPaCa-2 and AsPC-1 cells transfected with siHuR in absence or presence of oxaliplatin, revealed that knocking down HuR significantly inhibits the protein expression of BARD1 in non-treated and treated conditions (compare lanes 1 and 2; 3 and 4, Figure 2D and Figure S2c). Although significant, the impact on protein expression is not as robust as its effect on BARD1 mRNA expression; this could be attributed to the higher stability of the BARD1 protein [55]. In addition, treatment with the small molecule inhibitor of HuR, KH-3 [54], resulted in significant inhibition of BARD1 protein expression in two cell lines (Figure 2E). Together, using multiple strategies and in different contexts, we demonstrated that inhibition of HuR directly affects BARD1 protein expression in PDAC cells. 

### 3.5. HuR Does Not Directly Stabilize BARD1 mRNA 

**Figure 3:**
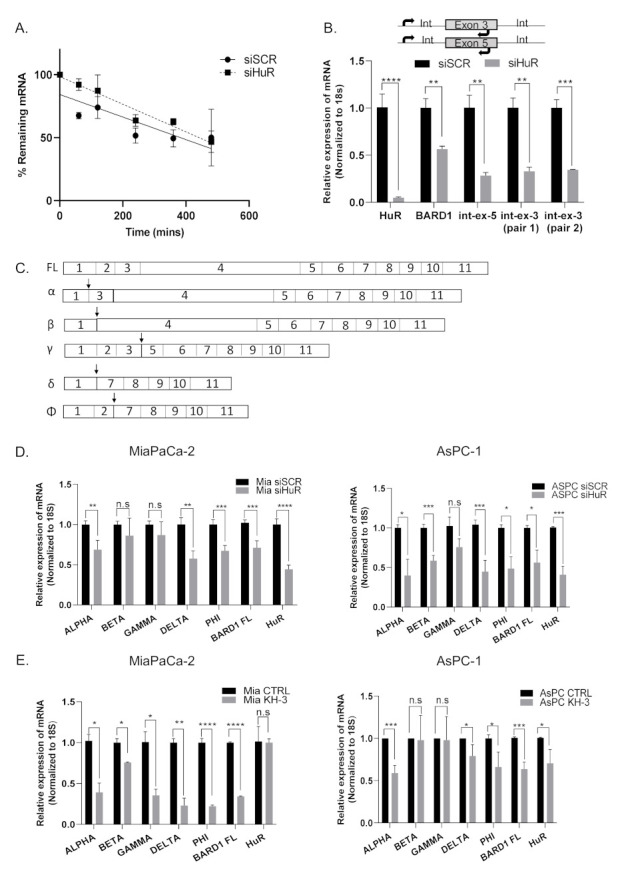
HuR regulates BARD1 pre-mRNA and isoform expression. (**A**) MiaPaCa-2 cells were transfected with siHuR (siHuR#1) and treated with actinomycin D (5 µg/mL) during the indicated times. BARD1 mRNA stability was assayed by RT-qPCR using GAPDH as a loading control. (**B**) RT-qPCR of BARD1 pre-mRNA using three different primer sets spanning intron/exon 5, and three in cells transfected with siHuR. Mean ± SEM, *n* = 3. **** *p* < 0.0001, *** *p* < 0.001, ** *p* < 0.01. (Student’s *t*-test). (**C**) Pictorial representation of BARD1 isoforms. Arrows indicate location of forward primer at specific exon junctions for each isoform. Reverse primers were located in the adjacent exon. Relative mRNA expression of BARD1 isoforms in MiaPaCa-2 and ASPC-1 cells transfected with either siHuR or siSCR (**D**) or treated with KH-3 (**E**). Mean ± SEM, *n* = 3. **** *p* < 0.0001, *** *p* < 0.001, ** *p* < 0.01, * *p* < 0.05, n.s, non-significant. (Student’s *t*-test).

Since silencing HuR decreased the levels of endogenous BARD1 mRNA, we analyzed whether HuR positively regulates BARD1 by stabilizing BARD1 mRNA. The half-life of BARD1 mRNA in siHuR cells compared with scrambled control was measured after de novo transcription was inhibited with treatment using the RNA polymerase II inhibitor actinomycin D, and the total RNA from siSCR- and siHuR-transfected cells were assessed over a period of time [15,16,17]. Importantly, we found that silencing HuR did not change the half-life of BARD1 mRNA (Figure 3A), suggesting that HuR does not stabilize BARD1 mRNA and may be regulating BARD1 mRNA by using an alternative mechanism. Knockdown efficiency of HuR is shown in Figure S3a.

### 3.6. HuR Regulates BARD1 in a Non-Canonical Manner

The most commonly cited mechanism by which HuR regulates its targets is the direct stabilization or destabilization of the target mRNA upon binding to 3′UTR ARE regions [10,15,16,17,56]. From publicly available datasets of HuR-RNP-IP, we found 12 putative AREs in the 3′UTR of BARD1 that span the entire region (Figure S3b), and we speculated that HuR binds these sequences in regulating the expression and stability of BARD1 mRNA. Apart from stabilizing mature mRNAs, HuR has also been found to regulate pre-mRNA or primary transcripts like other RBPs [57,58]. Since HuR effected mature BARD1 mRNA levels but did not influence its stability, we speculated that HuR might modulate the target earlier in its processing [58,59,60]. We first evaluated its effect on nascent transcription (i.e., levels of pre-mRNA) of BARD1. We compared the relative expression of newly transcribed BARD1 RNA by RT-qPCR using primer pairs that detect levels of nascent RNA prior to splicing. These primer pairs span intron–exon junctions such that one primer per pair aligned within an exon, and the other in the neighboring intron (Figure 3B). Total levels of the mature transcript were also evaluated using primer pairs spanning exon–exon junctions. Interestingly, silencing HuR significantly decreased both the pre-mRNA levels of BARD1 as well the mature mRNA (Figure 3B). As a negative control, HuR silencing did not alter the pre- or mature mRNA expression of GAPDH (Figure S3c), suggesting regulation of BARD1 mRNA abundance by HuR is an early event at the pre-mRNA stage, and perhaps regulates transcription.

Since HuR has previously been shown to regulate pre-mRNAs by binding to intronic sequences and to participate in alternative splicing events, we also evaluated if HuR regulates expression of BARD1 isoforms (Figure 3C), only some of which are protein coding with ORFs (NCBI RefSeq). In various cancer types, differential expression of these BARD1 isoforms (α, β, γ, δ, and Φ) has been demonstrated to carry out a pro-tumorigenic role [33,36,38,39,40]. We found that both forms of HuR inhibition (siHuR and KH-3) downregulated mRNA expression of multiple BARD1 isoforms (α, δ, Φ more consistently), suggesting that HuR regulates expression of BARD1 isoforms as well as the full-length (FL) mRNA transcript [54] (Figure 3D,E).

Furthermore, when we compared HuR-RNP-IP samples from control vs. olaparib treated cells, we found that HuR binds to isoform δ and Φ, suggesting a direct interaction of these isoforms like FL with HuR (Figure S3d). We were unable to confirm whether HuR stabilizes the protein levels of these isoforms as isoform specific antibodies are not available. 

### 3.7. BARD1 Supports PDAC Growth and Modulates Drug Responses

**Figure 4:**
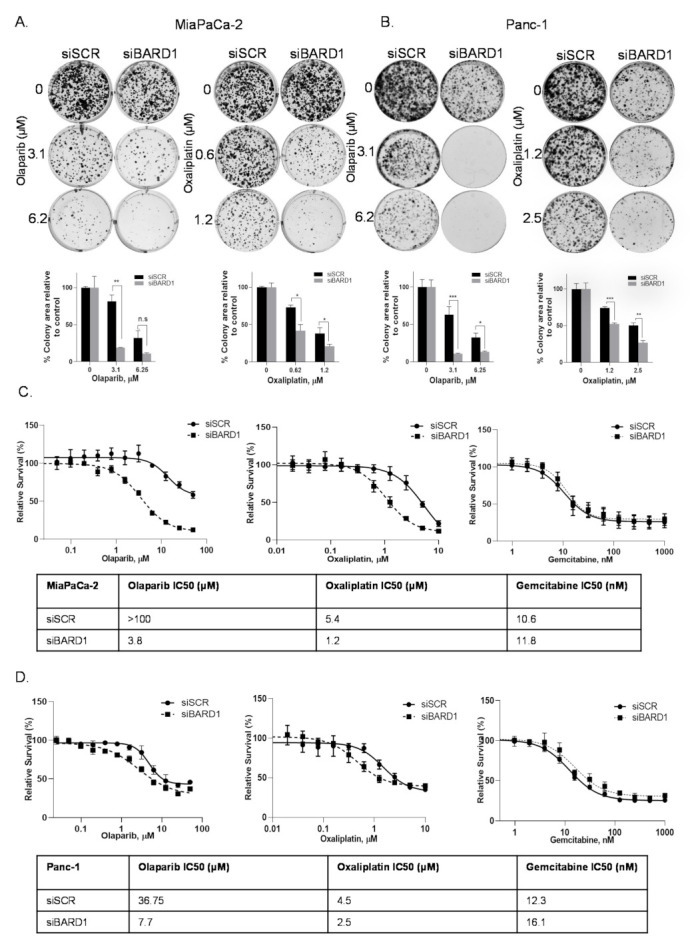
BARD1 supports PDAC growth and modulates drug responses. (**A**,**B**) Colony formation assays in BARD1 silenced (siBARD1#1), and MiaPaCa-2 and Panc-1 cells treated with either olaparib or oxaliplatin at indicated concentrations; colonies were stained with Coomassie blue after 14 days. Graphs of Mean ± SEM from *n* = 3 are shown. * *p* < 0.05, ** *p* < 0.01, *** *p* < 0.001, n.s, non-significant. (Student’s *t*-test). (**C**,**D**) Pico Green cell survival curves in BARD1 silenced (siBARD1#1), and MiaPaCa-2 cells and Panc-1 cells were treated with either olaparib, oxaliplatin, or gemcitabine for 5 days. Representative graphs from *n* = 3 are shown. IC50 doses are shown in the table.

It has been previously established that HuR’s stabilization of pro-survival mRNAs is an important facilitator of resistance to the cytotoxic effects of chemotherapy agents [10,15,16,17]. Here, we explored the role of BARD1 in DNA damage response after treating cells with olaparib and oxaliplatin [42]. Silencing BARD1 with two different siRNAs (siBARD1#1 and siBARD1#2) significantly increased the sensitivity of cells to olaparib and oxaliplatin, measured by both colony formation assay and pico green cell survival assay in both MiaPaCa-2 and Panc-1 cells (Figure 4A–D and Figure S4b–d). Relative mRNA expression of FL BARD1 in siBARD1 vs. siSCR is shown in Figure S4a). Note: we observed similar downregulation of all other isoforms at the mRNA level with use of these siRNAs (data not shown). A table of IC50 doses from the pico green cell survival assay is embedded in each graph. 

### 3.8. Knockdown of BARD1 Increases Drug-Induced DNA Damage 

**Figure 5:**
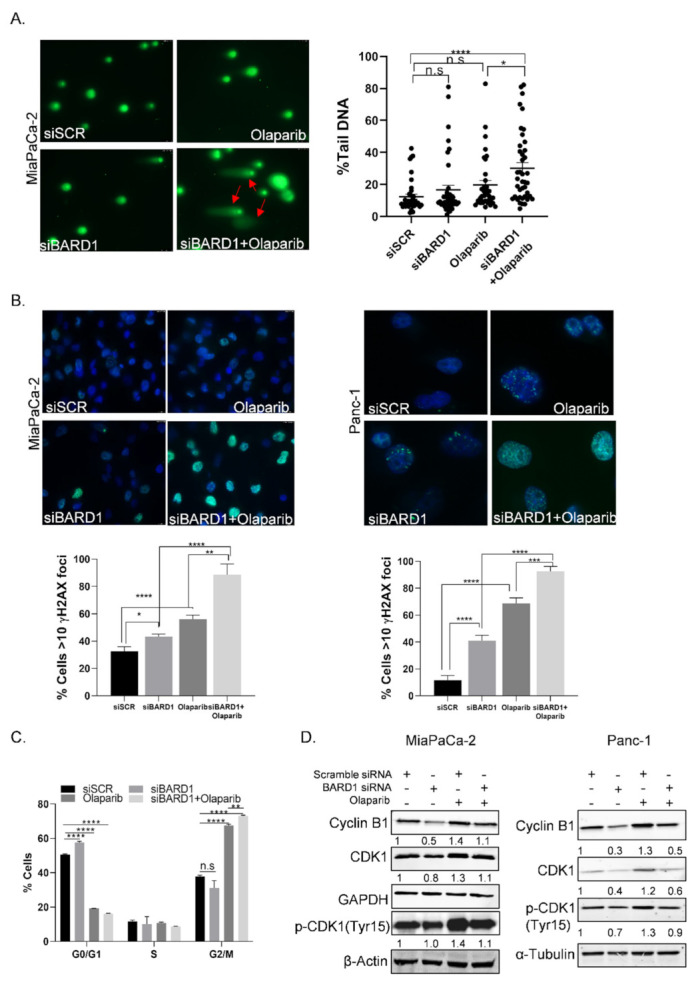
Knockdown of BARD1 increases drug induced DNA damage. (**A**) Comet assay showing an increase in olaparib (10 µM) induced DNA damage when cells were transfected with siBARD1 (siBARD1#1) as compared to siSCR control (the scale bar: 10–50 μM). Representative images from *n* = 3 and a graph of percentage Tail DNA is shown. Mean ± SEM. * *p* < 0.05, **** *p* < 0.0001, n.s, non-significant. (One-way ANOVA, Dunnett’s correction) (**B**) Immunofluorescence staining of γ-H2AX foci in siBARD1 (siBARD1#1) cells treated with or without olaparib (10 µM) for 24 h in MiaPaCa-2 and Panc-1 cells (the scale bar: 1–10 μM). Representative images from *n* = 3 are shown. Mean ± SEM. * *p* < 0.05, ** *p* < 0.01, *** *p* < 0.001, **** *p* < 0.0001 (Student’s *t*-test). (**C**) Graph showing different phases of the cell cycle as quantitated by flow cytometry and PI staining in MiaPaCa-2 cells transfected with siBARD1 (siBARD1#1) and treated with or without olaparib (10 µM) for 24 h. Mean ± SEM from *n* = 3. ** *p* < 0.01, **** *p* < 0.0001, n.s, non-significant (Student’s *t*-test). (**D**) Western blot analysis showing protein expression of cell cycle proteins, β-actin, GAPDH, α-tubulin in MiaPaCa-2, and Panc-1 cells. Representative blots from *n* = 3 are shown.

We performed comet assays and γ-H2AX foci staining to analyze the amount of double-strand breaks in presence of drugs, as well as the effect of silencing BARD1 on the degree of DNA damage. Cells were treated with or without olaparib (at 10 µM, the dose used for RNP-IP assays as in Figure 1), and we observed that silencing BARD1 (siBARD1#1) further conferred an increase in DNA damage as seen in the Comet assay by percentage Tail DNA, compared to siSCR control or olaparib alone (Figure 5A). We also detected an increase in γ-H2AX foci with combined BARD1 silencing and olaparib treatment in both MiaPaCa-2 and Panc-1 cells (Figure 5B). A statistically significant increase (*p* < 0.05 for MiaPaCa-2 and *p* < 0.0001 for Panc-1) in γ-H2AX foci was also seen in cells transfected with BARD1 siRNA as compared to siSCR (Figure 5B), indicating that BARD1 inhibition alone is sufficient to induce DNA damage.

To understand how the inhibition of BARD1 decreases cell survival and enhances drug induced DNA damage, we assessed cell-cycle changes when silencing BARD1 (siBARD1#1) in the absence or presence of drugs (Figure 5C). BARD1 knockdown alone induced cell cycle arrest at the G0/G1 phase, which was accompanied by a decrease in mitotic cyclin B1/CDK1 expression (Figure 5D), indicating a modulation of the cell cycle. Combined BARD1 silencing and olaparib treatment in MiaPaCa-2 cells caused a marked increase in G2/M arrest. This was also accompanied by an increase in *p*-CDK1 (tyr 15)/CDK1 ratio which illustrates increase in inhibitory phosphorylation of CDK1, and that cells are not progressing to mitosis (Figure 5D and Figure S5). 

### 3.9. Overexpression of BARD1 in HuR Silenced Cells Partially Rescues HRR Phenotype

**Figure 6:**
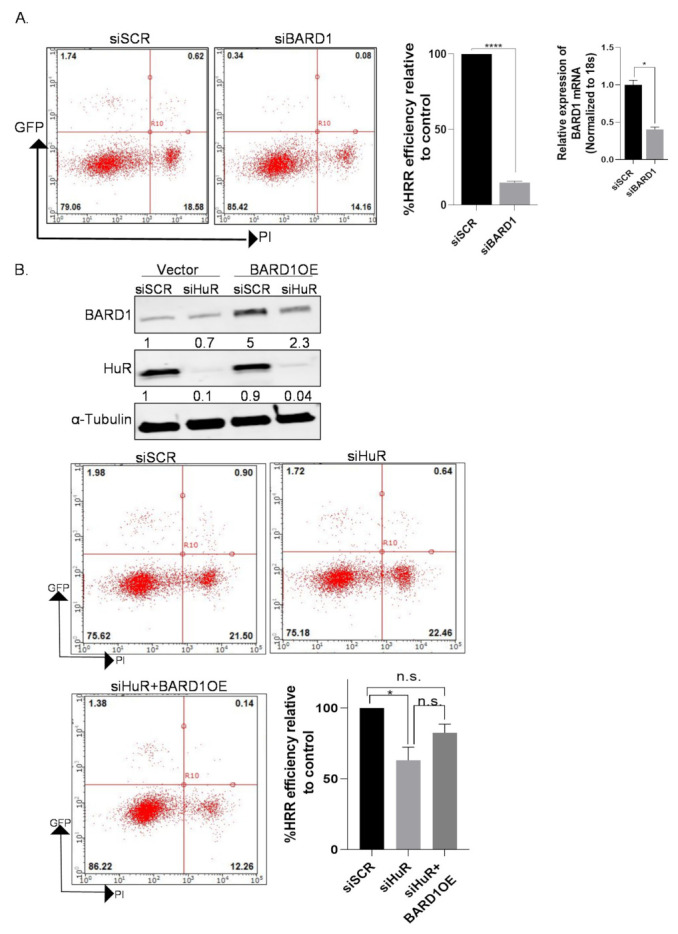
BARD1 partially rescues HuR’s HRR phenotype. (**A**) MiaPaCa-2-DRGFP cells were transfected with siBARD1, and 24 h later, re-transfected with pCBASceI (10 µg). Graph shows the percentage of HRR efficiency by comparing siSCR and siBARD1 calculated by quantitating GFP-positive cells by flow cytometry. * *p* < 0.05, **** *p* < 0.0001 (Student’s *t*-test). (**B**) pDR-GFP assay, graph, and western blot validation of BARD1-overexpression (BARD1OE) in MiaPaCa-2-DRGFP cells transfected with siHuR. * *p* < 0.05, n.s., non-significant.

To assess the direct impact of BARD1 on HRR efficiency, we silenced BARD1 (siBARD1#1) and assessed GFP positivity in the pDR-GFP assay [21,22]. MiaPaCa-2-DRGFP cells transfected with siBARD1 were re-transfected with pBASceI (Isce endonuclease) to induce damage, and 96 h later, GFP-positive cells were quantitated using flow cytometry. In siBARD1 cells, there was a significant decrease in HRR efficiency (>80%) compared with control (siSCR) cells (Figure 6A). These results suggest that BARD1 is vital for the homologous recombination repair of PDAC cells. To understand if BARD1 mediates the HuR-dependent HRR phenotype as seen in Figure 1, a plasmid encoding the full-length (FL) BARD1 cDNA was transfected in MiaPaCa-2-DRGFP cells that were previously transfected with siHuR (Figure 6B, upper panel). These cells were then analyzed to determine if the exogenous expression of BARD1 rescues the DNA damage phenotype (loss of HRR efficiency) seen when silencing HuR in PDAC cells. We found that an overexpression of BARD1 did “partially” rescue the HRR phenotype in HuR silenced cells (Figure 6B, lower panel). The rescue was only partial and did not completely reach baseline levels, possibly because knockdown of HuR may negatively regulate not only BARD1, but also other proteins involved in DNA repair, which would affect the rescue efficiency after BARD1 overexpression. To investigate the specificity of the BARD1 rescue, we assessed the impact of BARD1 overexpression in an established HuR-driven phenotype [11,12,41]. We have previously reported that HuR knockout (Mia −/−) cells display a slow growing phenotype [11,41]; therefore, we performed a colony formation assay to assess if BARD1 overexpression was also able to rescue this effect. As shown in Figure S6a,b, exogenous expression of BARD1 did not, to any extent, rescue the slower growth of HuR knockout cells, either in the absence or presence of drug treatments, suggesting that the HuR-BARD1 axis is most likely specific to the HRR functions of PDAC cells, independently of other HuR pro-oncogenic functions [12].

## 4. Discussion

In this study, we elucidated a direct interaction between the RBP, HuR, and BARD1 mRNA, demonstrating that HuR post-transcriptionally promotes BARD1 expression in PDAC cells (Figure 1 and Figure 2). This regulation of HuR-BARD1 could: (1) work in favor of PDAC cells undergoing transient DNA damage and prevent further accumulation of DNA lesions; (2) impart resistance to DNA damaging agents by facilitating an important repair network; and/or (3) help genomic stable/repaired cells to invade and metastasize at distant organs [29,61,62]. Previous reports have established that HuR negatively regulates translation of the BRCA1 protein with no significant effect on mRNA expression or its stability in HeLa cells [56]. Another study established that HuR increased the mRNA expression of the RAD51-associated protein1 (*RAD51AP1*) mRNA in breast cancer cells [63]. However, we did not detect significant binding or changes in these HRR genes in our screen, suggesting that differences in HuR biology could be dependent on cancer cell type. We also looked at the gene signature from the NHEJ (non-homologous-end-joining) pathway using siHuR-RNA-seq data and found no significant de-enrichment of this pathway upon HuR knockdown (data not shown), suggesting that the HuR-BARD1 axis is specifically an important transient regulatory hub of the HRR pathway in PDAC cells. It is possible that HuR could also potentially facilitate other DNA repair pathways by modulating genes other than BARD1, as previously shown by our group [15,16]. 

Upregulation of the cytoplasmic expression of BARD1 has been found in breast, ovarian, non-small cell lung, and hepatocellular cancers [33,34,35,40,64]. Pathogenic variants of BARD1 have also been reported in PDAC patients with or without a family history of PDAC [51,52,53]. However, the mechanism(s) behind BARD1 regulation, and its mRNA abundance in PDAC, is currently unknown. Previous studies have shown that BARD1 expression in cells could be controlled by a balance of post-transcriptional regulation by long non-coding RNAs (lncRNAs) and miRNAs [65]. Here, we show for the first time that BARD1 expression in PDAC cells is regulated by an RBP, HuR. This raises the intriguing question of whether HuR competes and/or cooperates with miRNAs/lncRNAs in regulating the expression of BARD1. 

Unlike many other targets of HuR [10,11,16], we found that HuR did not stabilize BARD1 mRNA levels, as the decay profile of BARD1 mRNA was similar in control versus HuR silenced cells (Figure 3A). This suggested that either other RBPs, non-coding RNAs, competing miRNAs that bind to similar ARE in the 3′UTR of BARD1 mRNA (Figure S3b), or the HuR regulation of BARD1 mRNA abundance, play a possible role at an earlier stage of mRNA processing, through direct or indirect transcription regulation. Through RT-qPCR studies evaluating nascent transcript expression, we found that HuR modulates levels of both BARD1 pre-mRNA and mature RNA, and subsequently upregulates protein expression in PDAC cells (Figure 3B). Alternative splicing by HuR has been reported through its binding affinity to conserved U-/AU-rich elements in the introns of its target transcripts, and we speculated that because HuR regulates pre-mRNA levels of BARD1, it could affect BARD1 mRNA splicing and further regulation of isoforms [26,27,50,57]. Indeed, in cells where HuR was inhibited, we found a significant decrease in the mRNA expression of some known BARD1 isoforms (significant changes were seen in isoforms α, δ, Φ, Figure 3D,E). This is important since many studies have highlighted the role of these alternatively spliced isoforms of BARD1 in the tumorigenesis of other cancer types [33,34,36,40,64,66]. Through our RNP-IP analysis, we found that HuR binds not only the FL, but also the δ, Φ isoforms (Figure S3d). We believe that because these isoforms share similar 3′UTR (NCBI Refseq databases, accession # NM_000465.4, NM_001282545.2, NM_001282543.2, NM_001282548.2), HuR binds the putative binding sequences (shown in Figure S3b) in both the isoforms and FL BARD1. FL BARD1 has been reported to possess tumor suppressor properties, whereas BARD1 isoforms have been reported to be pro-oncogenic in many tumor types, antagonizing the functions of FL, indicating that there is a balance between the expression of oncogenic isoforms of BARD1 and FL BARD1 [32,34,36,37,38,64]. How HuR participates in splicing events at the pre-mRNA level to regulate different BARD1 isoform expression, and how it maintains this balance, is being investigated in ongoing studies. 

In this study we also found that BARD1 is necessary for both PDAC cell survival (Figure 4 and Figure 5) and DNA homologous recombination repair (HRR). We found that BARD1 downregulation enhanced the DNA damage response of olaparib in PDAC cells by arresting cells in G2/M phase of the cell cycle. BARD1 inhibition alone caused an increase in γ-H2AX foci, a marker of DNA damage, and loss of homologous recombination repair, indicating that BARD1 loss is toxic to PDAC cells. We did not observe a similar significance with comet assays (siSCR vs. siBARD1), possibly pointing to differences in sensitivities of the two assays. The siRNAs that were used in this study to transiently inhibit BARD1 targeted both FL and isoforms of BARD1 (data not shown), and hence, it will be interesting to understand which form of BARD1 drives the phenotype seen in PDAC cells. We also demonstrated that BARD1 is an essential mediator of the HuR-induced HRR response, as an overexpression of full-length (FL) BARD1 partially rescued the repair deficient, but not the slow-growing phenotype of HuR-silenced cells (Figure 6 and Figure S6) [12,13,30,67].

## 5. Conclusions

In conclusion, we demonstrate for the first time that BARD1 mRNAs (i.e., isoforms) are targets of HuR in PDAC. HuR’s post-transcriptional gene regulation of BARD1 may be a key regulatory point in the acute, pro-survival HRR pathway in PDAC cells. Thus, future studies will attempt to disrupt the HuR-BARD1 mRNA interaction in an effort to enhance current DNA damaging agent-based therapies, as well as to identify novel ones.

## Data Availability

The RNA-seq and RNP-IP microarray datasets supporting this article are available in the NCBI Gene Expression Omnibus (GEO), under the accession numbers # GSE167525 and #GSE166951. All other data generated or analyzed during this study are included in this published article.

## Supplementary Materials

The following supporting information can be downloaded at: https://www.mdpi.com/article/10.3390/cancers14071848/s1, Figure S1: HuR regulates homologous recombination repair in PDAC cells and BARD1 Oncomine and Survival data; Figure S2: HuR regulates mRNA and protein expression of BARD1; Figure S3: HuR regulates BARD1 isoform expression and BARD1 3′UTR binding sites; Figure S4: BARD1 supports PDAC growth and modulates drug responses; Figure S5: Knockdown of BARD1 increases pCDK1/CDK1 ratio in combination with olaparib; Figure S6: BARD1 does not rescue HuR’s growth phenotype; Figure S7: original blots; Table S1: GSEA details from Reactome HRR siHuRVSsiSCR; Table S2: Top differentially expressed genes HuR vs. IgG RNP-IP; Table S3: Log2FC changes in Reactome HRR genes in RNA seq and RNP-IP assays.
