# Supplementary material for: HuR Plays a Role in Double-Strand Break Repair in Pancreatic Cancer Cells and Regulates Functional BRCA1-Associated-Ring-Domain-1(BARD1) Isoforms
Source: Cancers (Basel). 2022 Apr 6;14(7):1848. doi: 10.3390/cancers14071848 (PMC8997573; doi:10.3390/cancers14071848)
Supplement: Supplementary file 1 [file cancers-14-01848-s001.zip › cancers-1604757-supplementary Figures S1-S6.pdf]

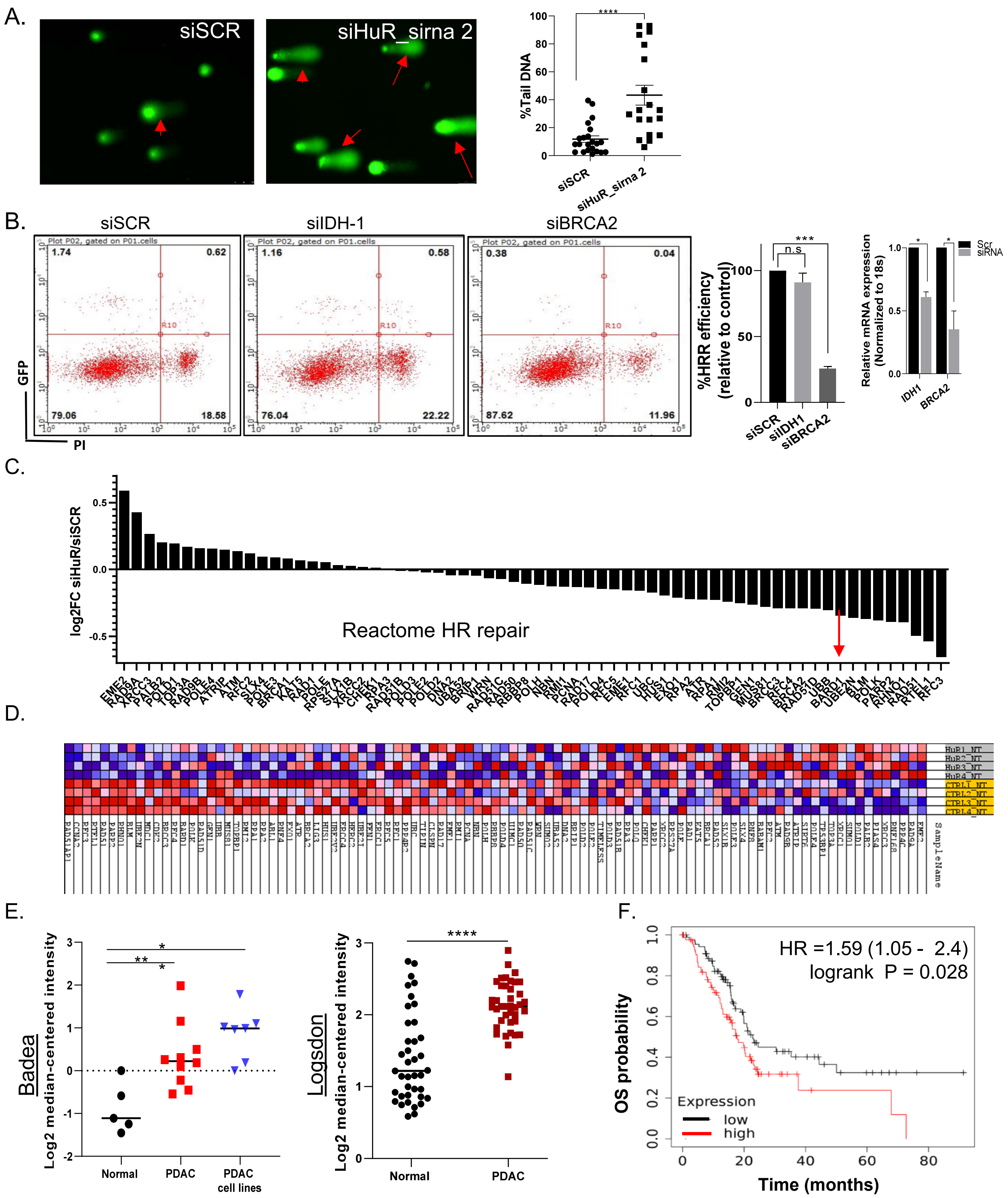

Figure S1. HuR regulates homologous recombination repair in PDAC cells and BARD1 oncomine and survival data. A) Comet assay showing increase in %Tail DNA in cells transfected with siHuR (siHuR#2) compared to siSCR. Mean ±SEM, n=3. ****p<0.0001. B) pDR-GFP assay in MiaPaCa-2-DRGFP cells transfected with either siSCR or siIDH-1 or siBRCA2 siRNAs and then re-transfected with pCBASceI (10 µg). Graph shows %HRR efficiency as calculated by quantitating GFP+ve cells by flow cytometry. Mean ±SEM, n=3. ***p<0.001, n.s, non-significant. C) Log2FC of genes from the Reactome HR Repair in siHuR (HuR_NT) versus siSCR (CTRL_NT) from n=4. D) Heatmap of genes from the Reactome HR repair MsigDB gene set in siHuR (HuR_NT) versus siSCR (CTRL_NT) as analyzed by GSEA. Red to blue shows range of expression values (high to low) E) Oncomine datasets (Badea and Logsdon) were statistically reanalyzed using two-sample t-tests, showing *BARD1* mRNA expression in normal versus PDAC cells/cell lines. ****p<0.0001, **p<0.01. F) Kaplan-Meir plot demonstrating overall survival probability in low versus high *BARD1* mRNA expression.HR=hazard ratio.

A.

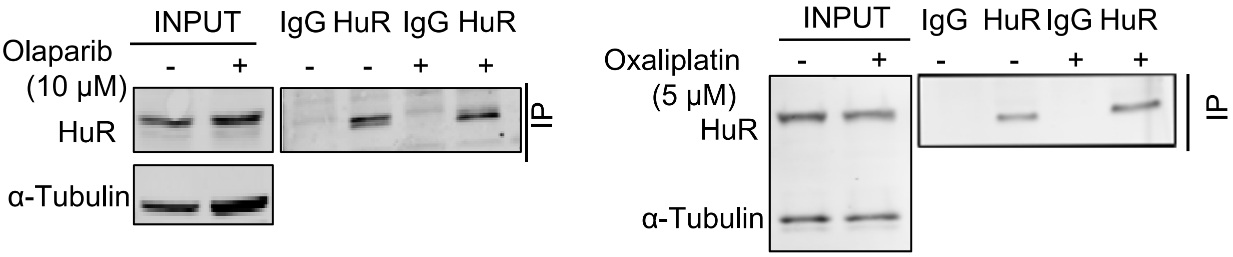

B.

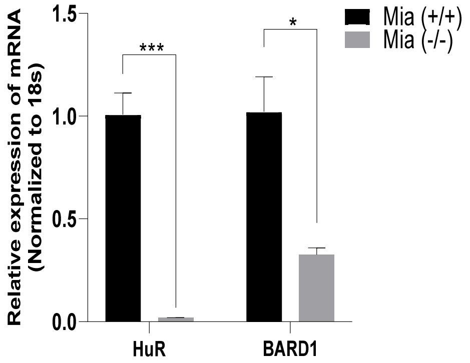

C.

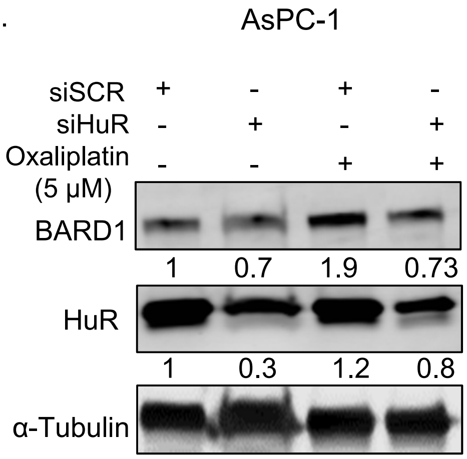

Figure S2. HuR regulates mRNA and protein expression of BARD1. A) Western blot validation of cytoplasmic fractionation and HuR-RNP-IP in olaparib (10 µM) or oxaliplatin (5 µM) treated MiaPaCa-2 cells. B) Relative expression of mRNA in Mia (+/+) and Mia (-/-) cells normalized to 18s. Mean ±SEM, n=3. ***p<0.001, *p<0.05. C) Western blot analysis of protein expression of BARD1, HuR and α-tubulin in AsPC-1 cells treated with oxaliplatin and transfected with siHuR (siHuR#1) for 48 hours.

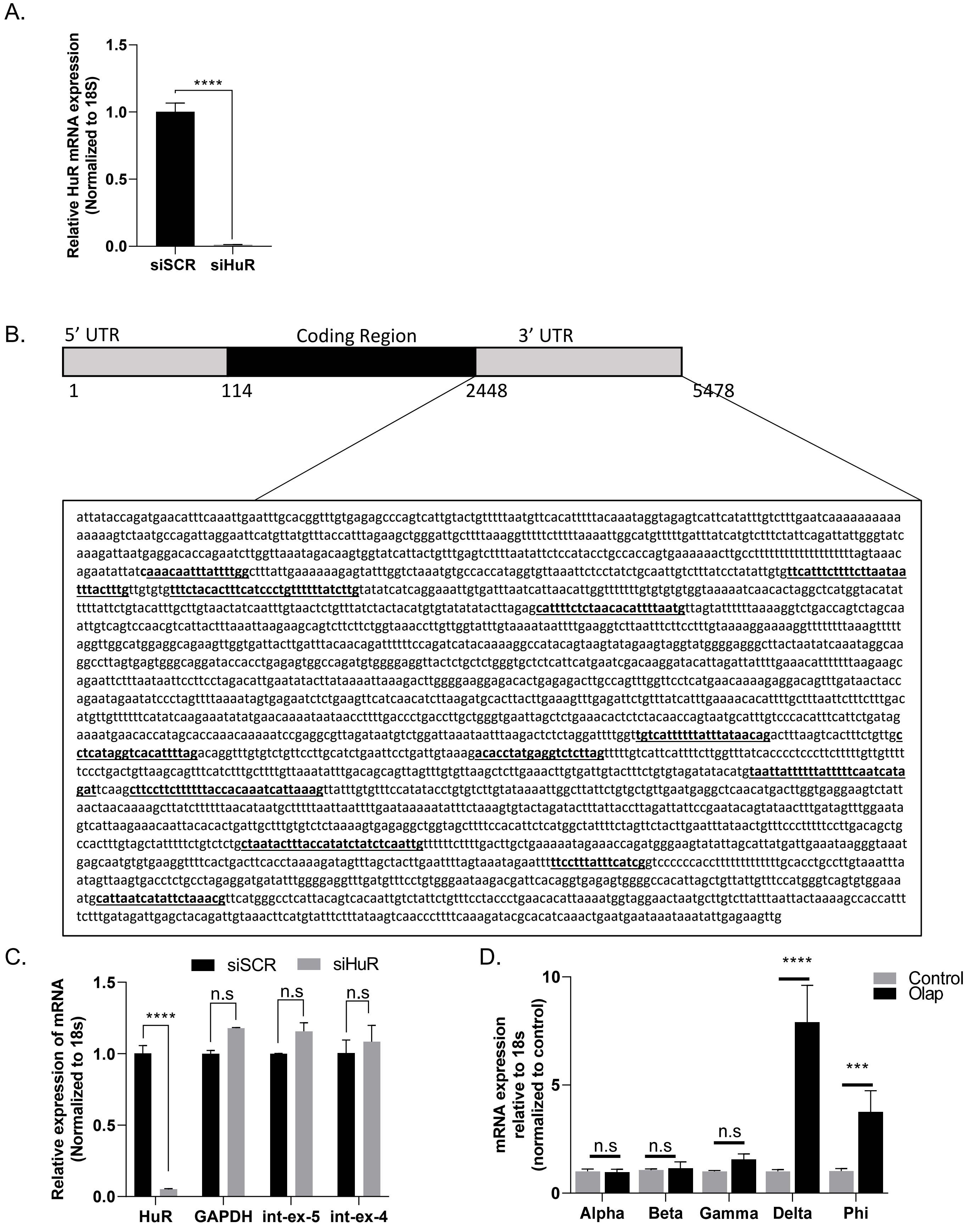

Figure S3. HuR regulates BARD1 isoform expression and BARD1 3'UTR binding sites. A) Relative mRNA expression of *HuR* after transfecting cells with siSCR or siHuR for Actinomycin D assay, as analyzed by RT-qPCR assay. Mean ±SEM, n=3. ****p<0.0001. B) Schematic of the 3'UTR sequence of BARD1. Putative HuR binding sites in 3'UTR of BARD1 are highlighted in bold and underlined**.** C) Relative mRNA expression of *GAPDH* (pre- and mature) in siSCR and siHuR (siHuR#1) transfected MiaPaCa-2 cells. Mean ±SEM, n=3.****p<0.0001, n.s, non-significant. D) Relative mRNA expression of BARD1 isoforms in MiaPaCa-2 HuR RNP-IP samples. Mean ±SEM, ****p<0.0001 ,***p<0.001, n.s, non-significant.

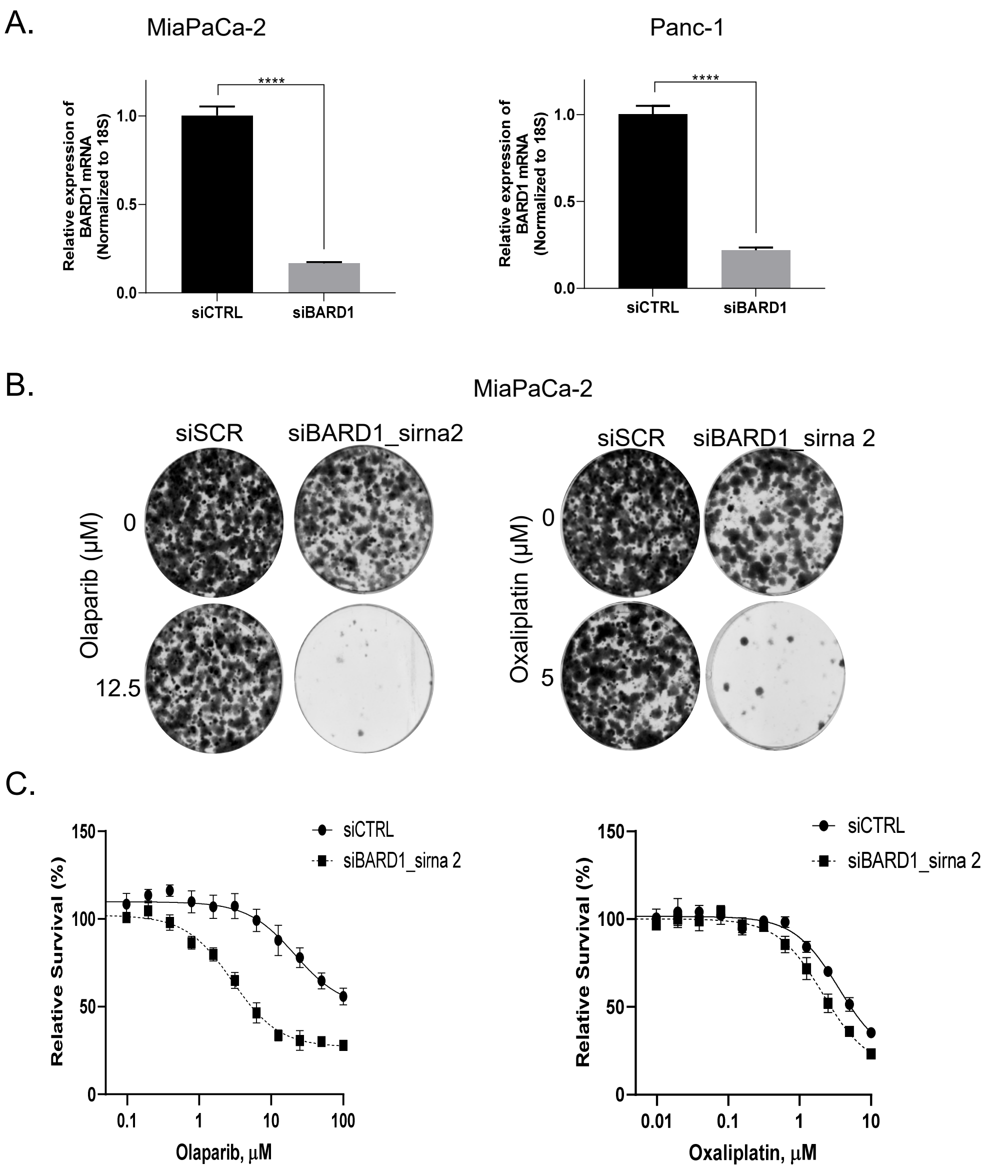

MiaPaCa-2	Olaparib IC50 (μM)	Oxaliplatin IC50 (μM)
siSCR	>100	7.4
siBARD1_sirna2	8.8	2.8

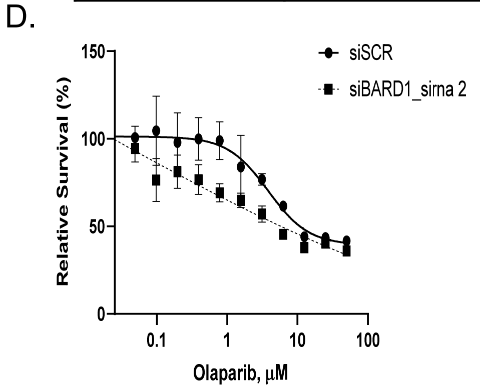

Panc-1	Olaparib IC50 (μM)
siSCR	26
siBARD1_sirna2	6.23

Figure S4. BARD1 supports PDAC growth and modulates drug responses. A) Relative expression of *BARD1* mRNA in MiaPaCa-2 and Panc-1 cells after transfection with BARD1 siRNA (siBARD1#1). Mean ±SEM, n=3. ****p<0.0001. B) Colony formation assays in BARD1 silenced (siBARD1#2) MiaPaCa-2 cells treated with olaparib or oxaliplatin. Colonies were stained with commassie blue after 14 days. C) and D) Pico Green assays in MiaPaCa-2 and Panc-1 cells after transfection with BARD1 siRNA (siBARD1#2). A table of IC50 is shown below.

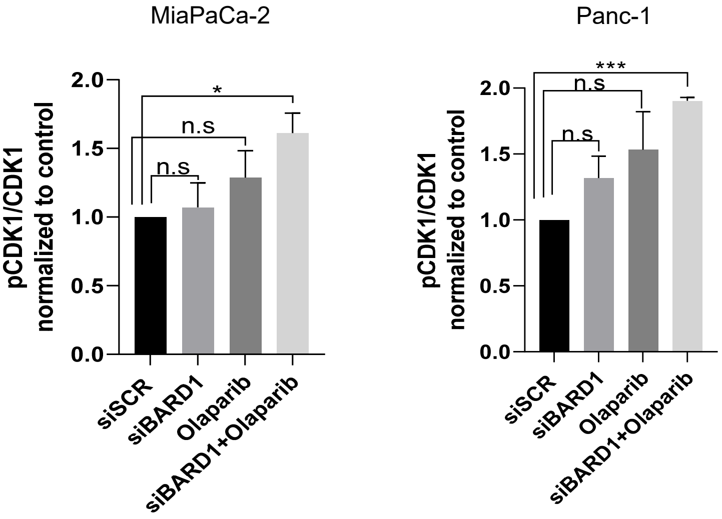

Figure S5. Knockdown of BARD1 increases pCDK1/CDK1 ratio in combination with olaparib. A) Graph of pCDK1/CDK1 normalized to control in MiaPaCa-2 and Panc-1 cells transfected either with siSCR or siBARD1 (siBARD1#1) and treated or untreated with olaparib (10 μM). Mean ±SEM, n=3. ***p<0.001, *p<0.05, n.s, non-significant.

A.

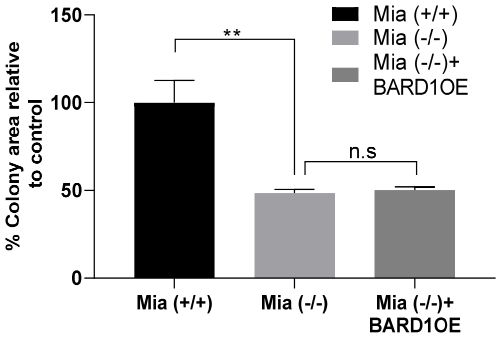

B.

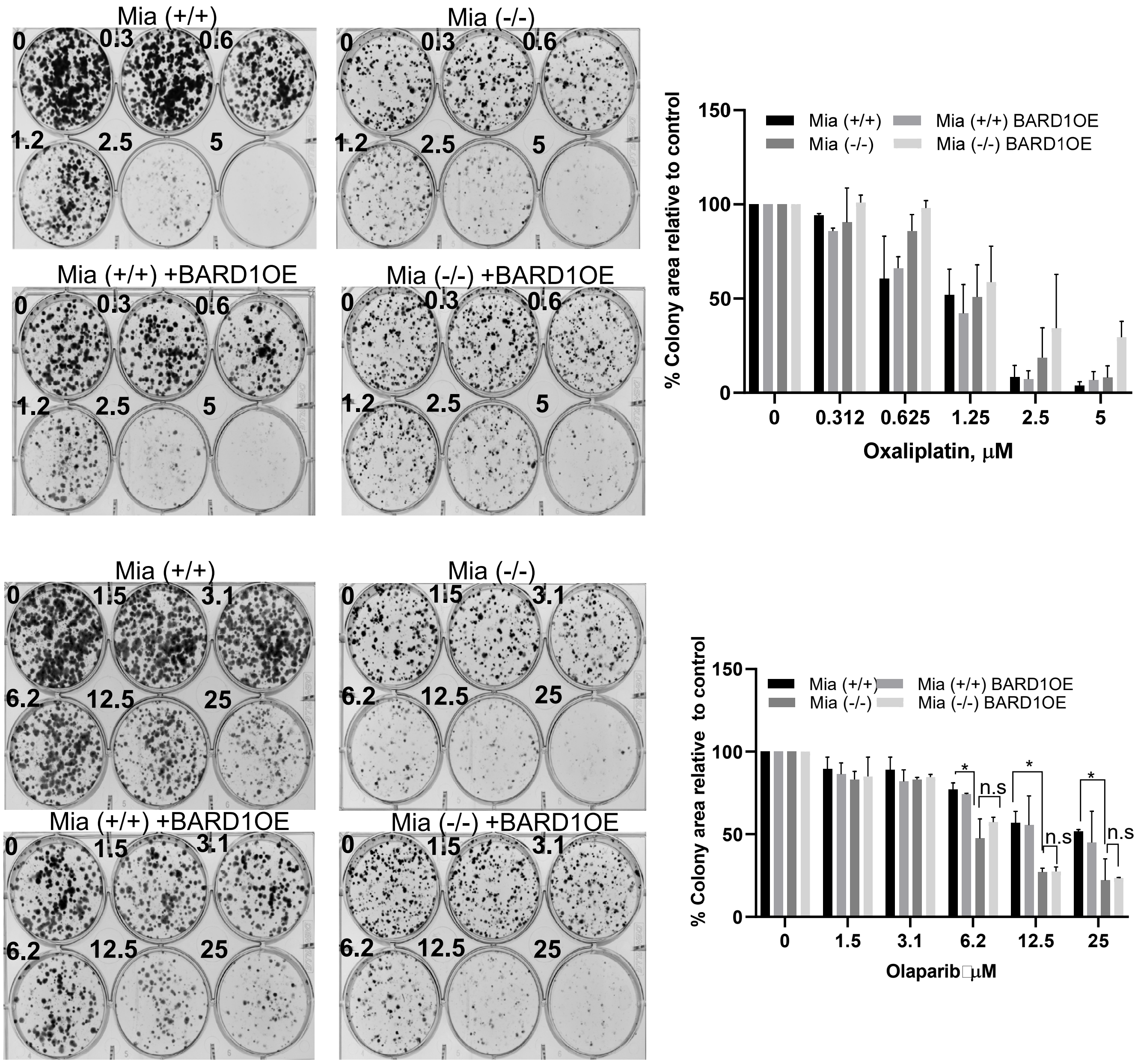

Figure S6. BARD1 doesnot rescue HuR's growth phenotype. Colony formation assays in Mia (+/+) and Mia (-/-) cells, where BARD1 is exogenously overexpressed (BARD1OE)**.** A) Graph of % colony area relative to control in Mia (+/+), Mia (-/-) and Mia (-/-) BARD1 overexpressed cells. Mean ±SEM, n=3. **p<0.01, n.s, non-significant. B) Colony formation images (left) and representative graphs (right) in oxaliplatin or olaparib treated Mia (+/+) or (-/-) cells. Mean ±SEM, n=3. *p<0.05, n.s, non-significant.
