# Supplementary figures and images for: HuR Plays a Role in Double-Strand Break Repair in Pancreatic Cancer Cells and Regulates Functional BRCA1-Associated-Ring-Domain-1(BARD1) Isoforms
Source: Cancers (Basel). 2022 Apr 6;14(7):1848. doi: 10.3390/cancers14071848 (PMC8997573; doi:10.3390/cancers14071848)

Fig 1d

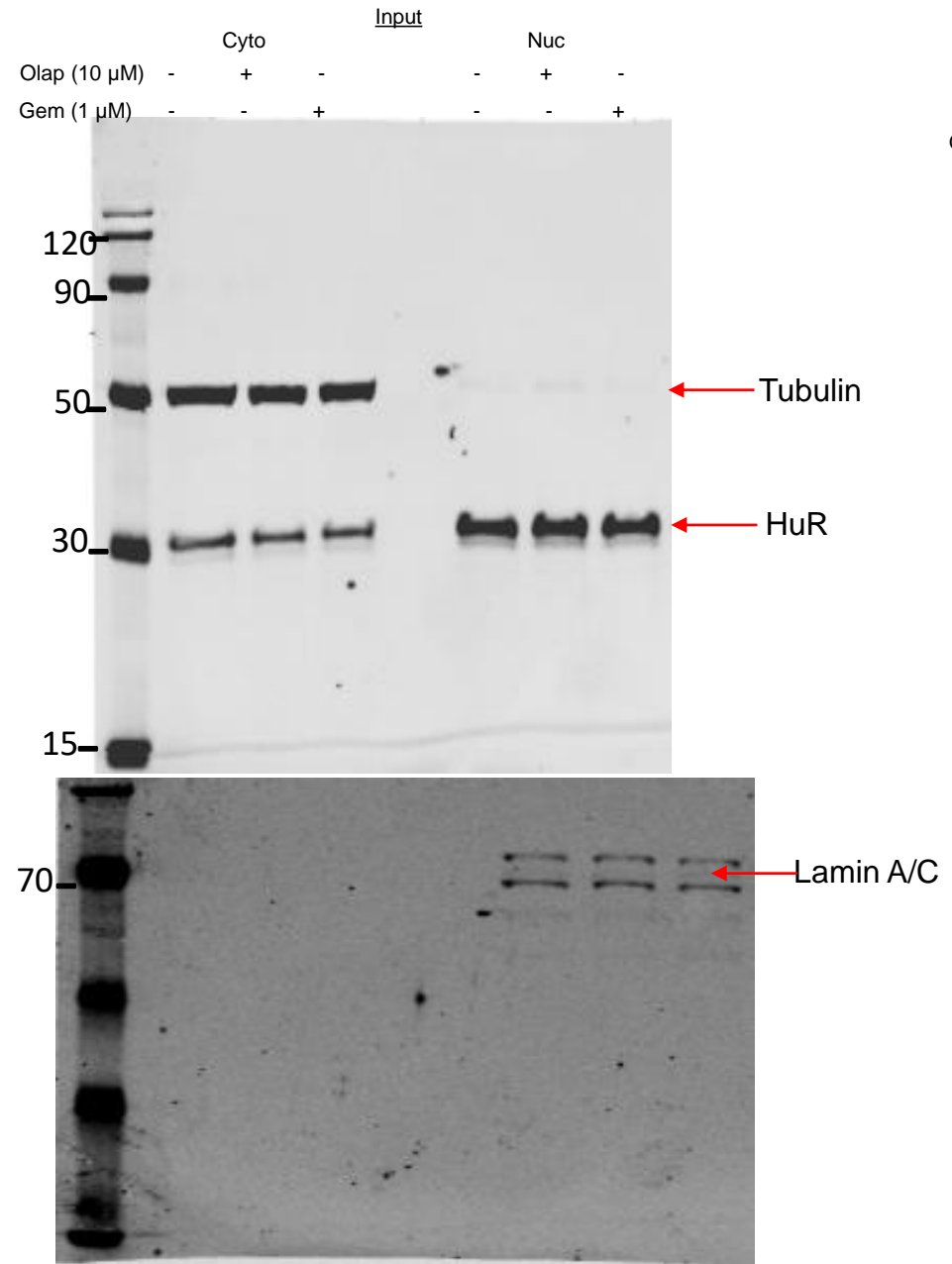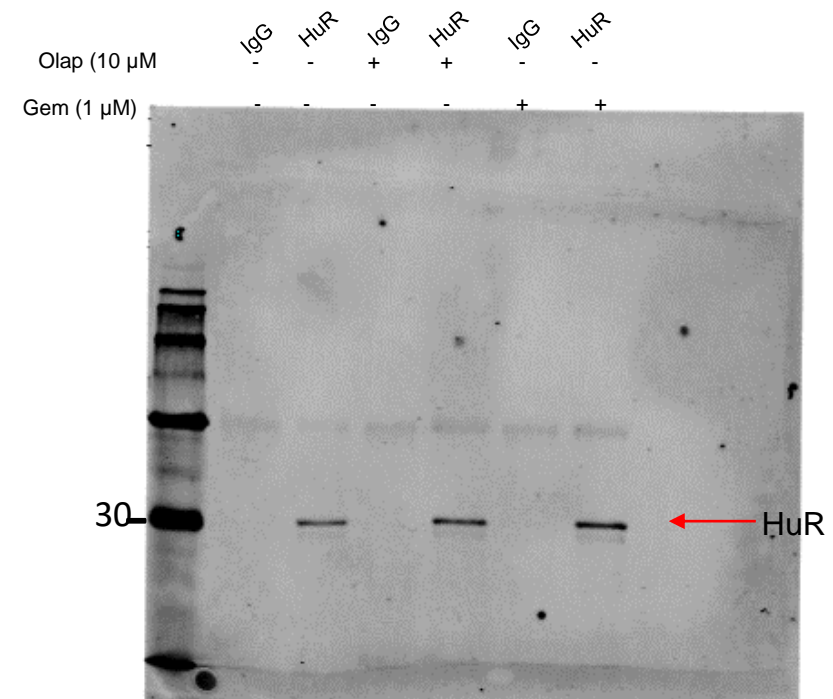

Fig 2d.

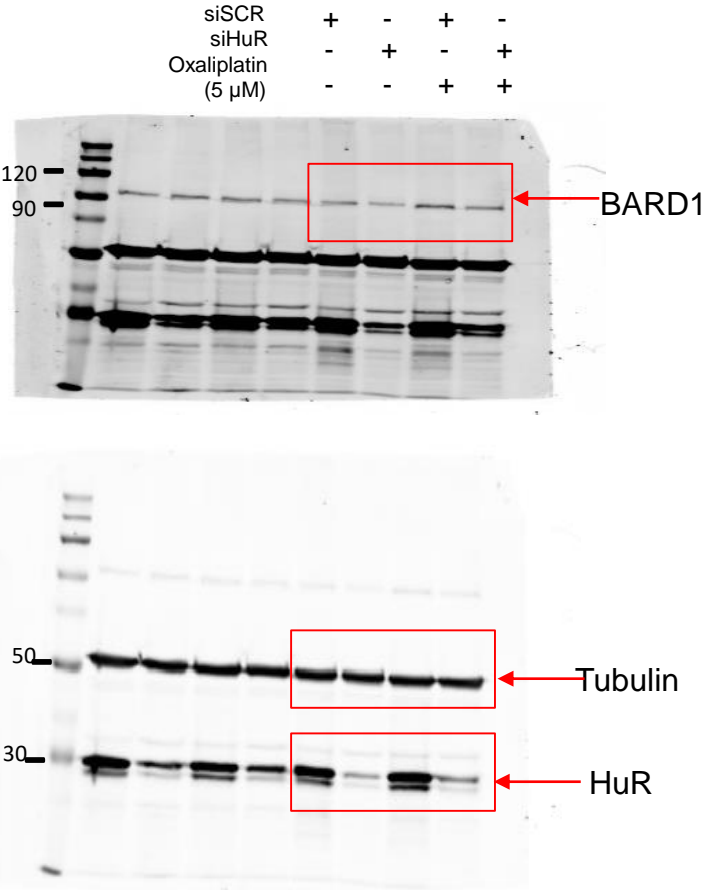

Fig 2e.

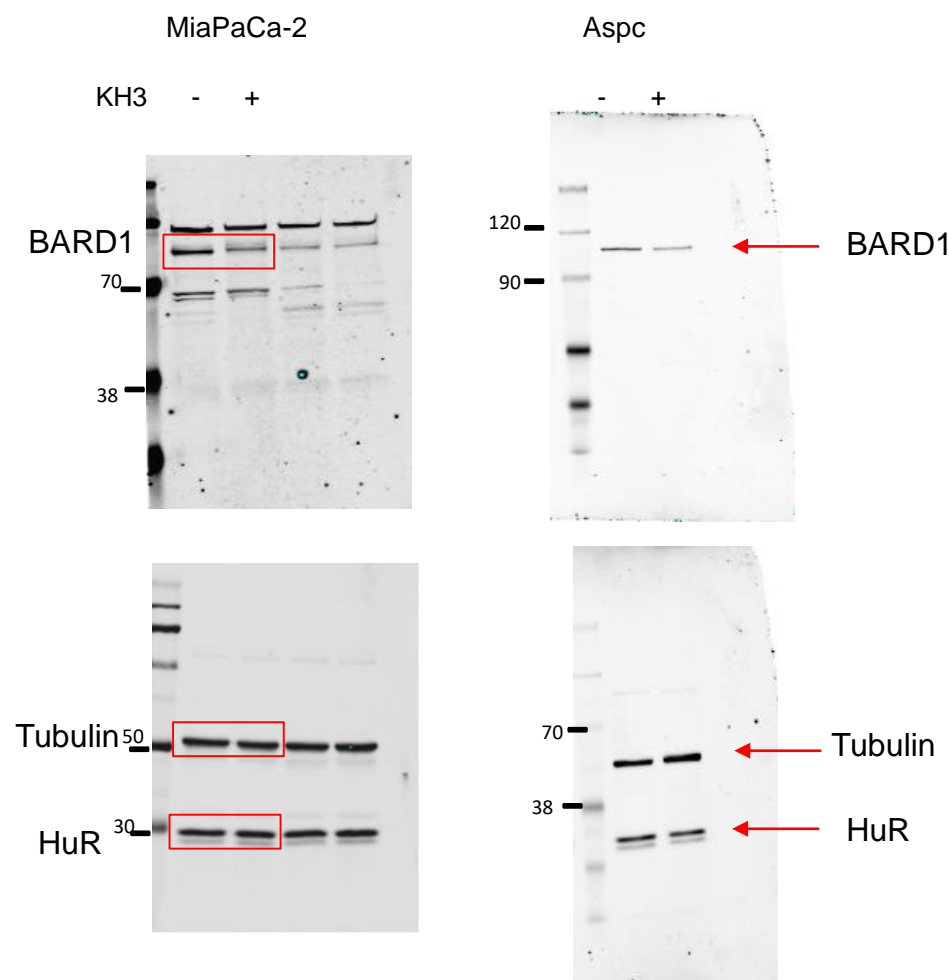

Fig S2a

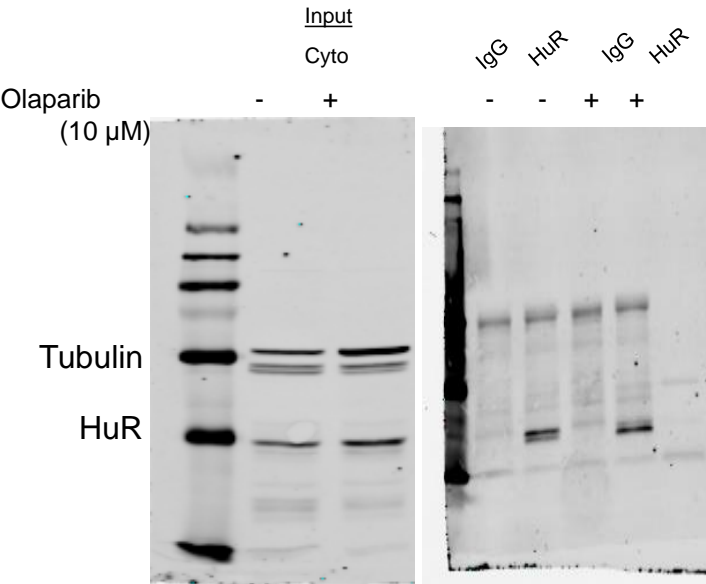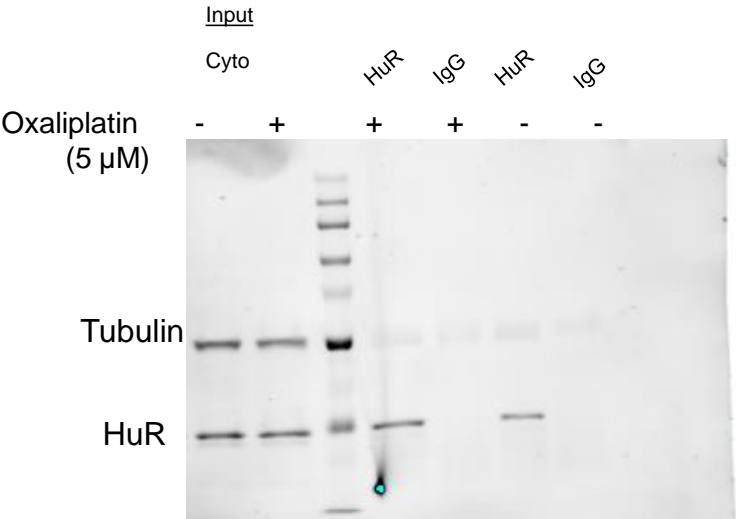

Fig S2c.

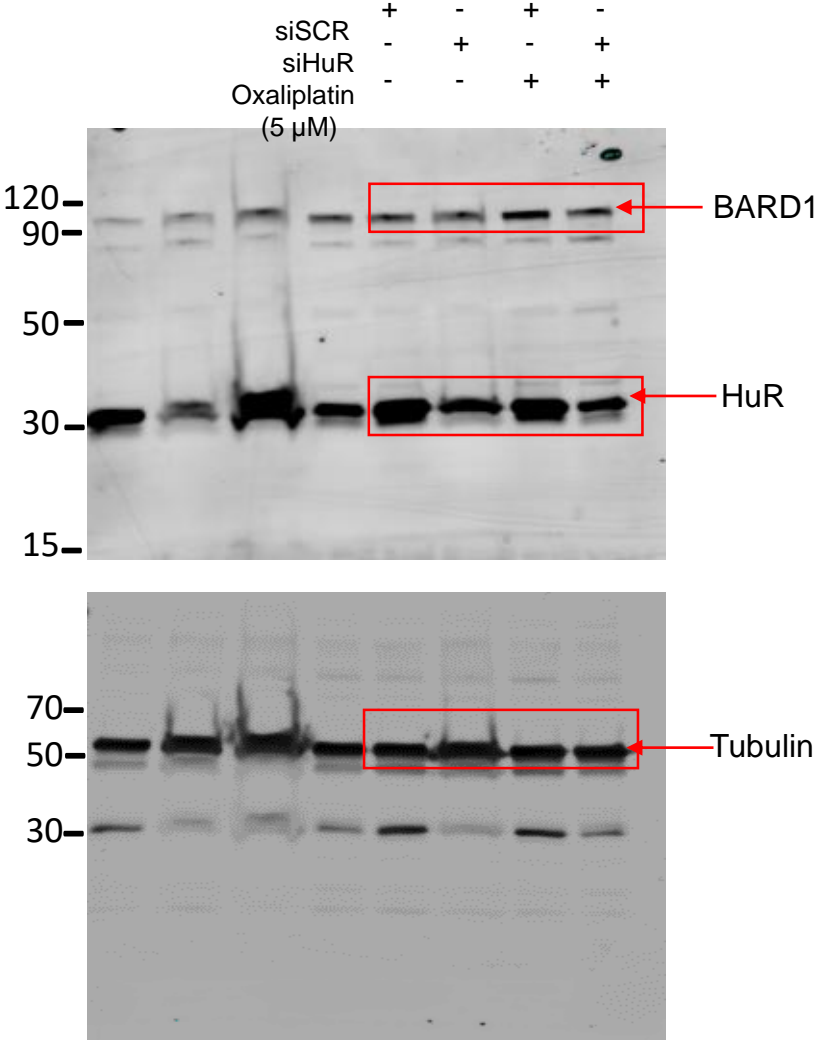

Fig 5D

MiaPaCa2

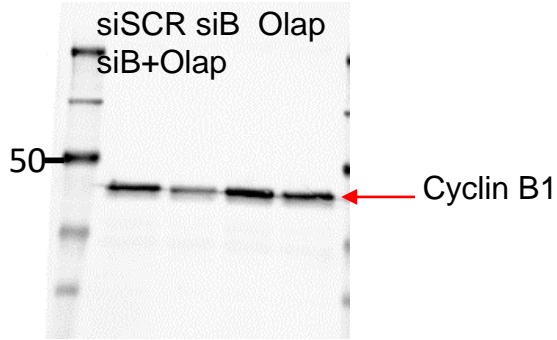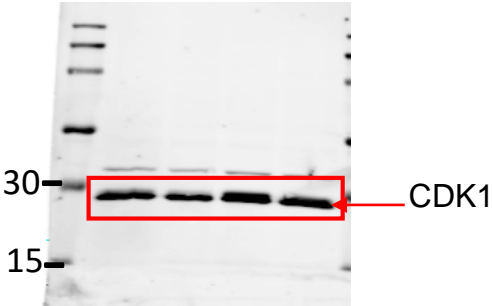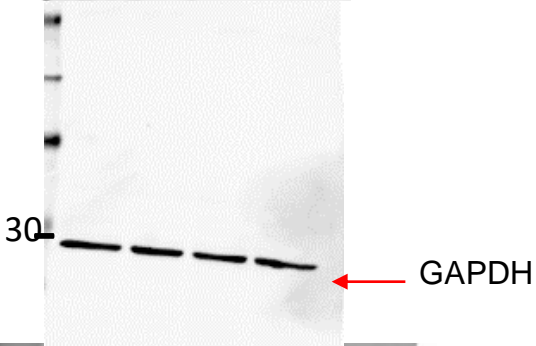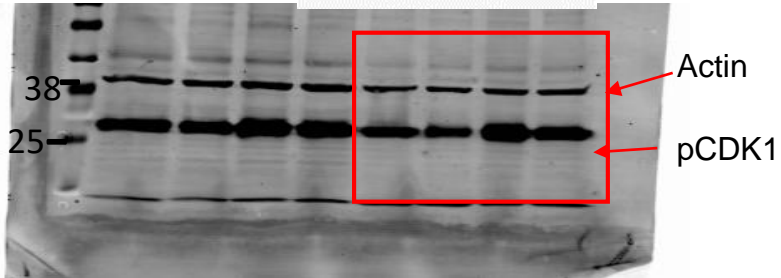

Panc-1

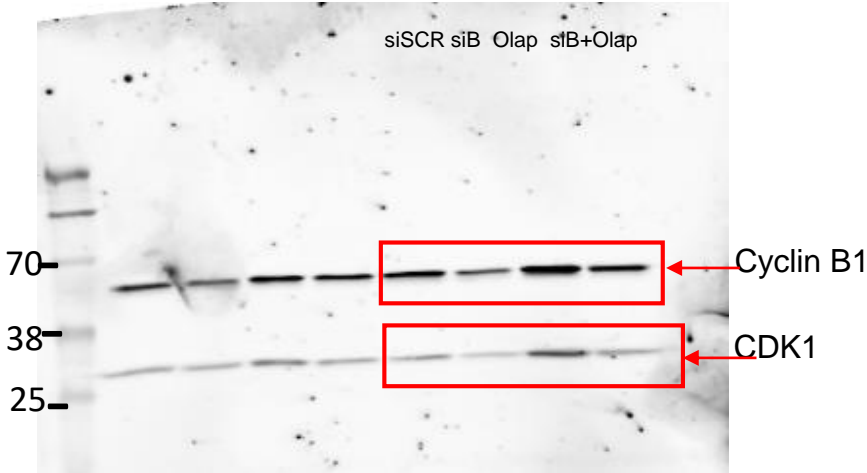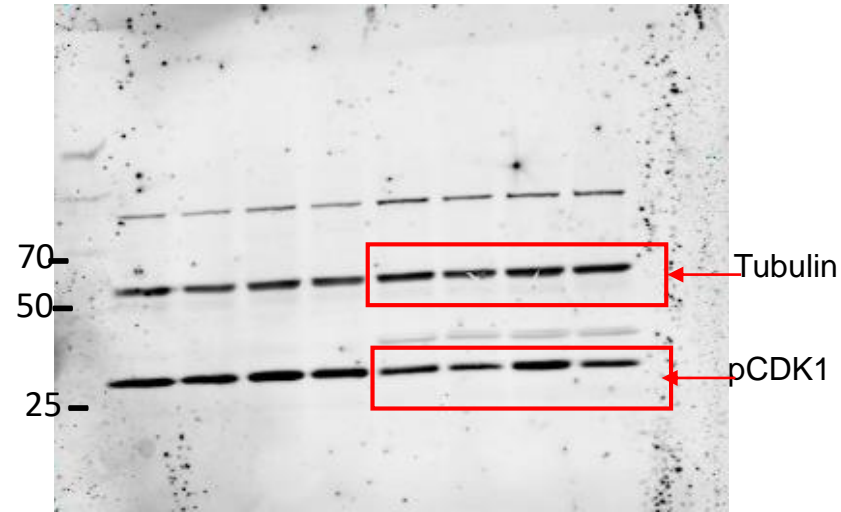

Figure 6b

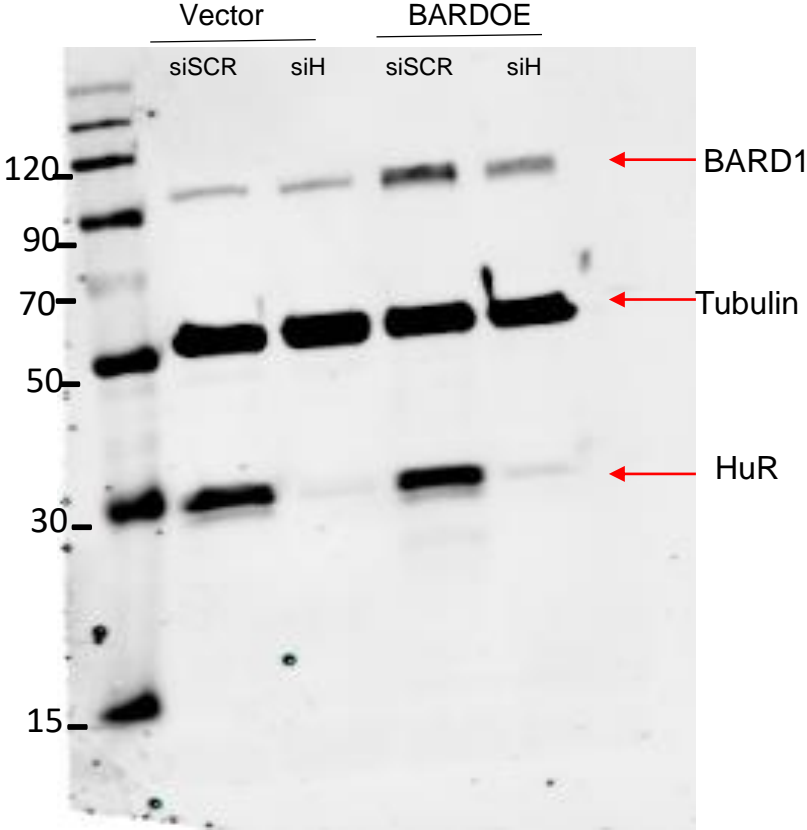

Supplement: Supplementary file 1 [file cancers-14-01848-s001.zip › cancers-1604757-supplementary Figure S7.pdf]
